# High-fidelity structured illumination microscopy by point-spread-function engineering

**DOI:** 10.1038/s41377-021-00513-w

**Published:** 2021-04-01

**Authors:** Gang Wen, Simin Li, Linbo Wang, Xiaohu Chen, Zhenglong Sun, Yong Liang, Xin Jin, Yifan Xing, Yaming Jiu, Yuguo Tang, Hui Li

**Affiliations:** 1 Jiangsu Key Laboratory of Medical Optics, CAS Center for Excellence in Molecular Cell Science, Suzhou Institute of Biomedical Engineering and Technology, Chinese Academy of Sciences, Suzhou, Jiangsu 215163 China; 2 Academy for Engineering and Technology, Fudan University, Shanghai, 200433 China; 3 The Center for Microbes, Development and Health, Key Laboratory of Molecular Virology and Immunology, Institute Pasteur of Shanghai, Chinese Academy of Sciences, Shanghai, 200031 China

**Keywords:** Imaging and sensing, Biophotonics

## Abstract

Structured illumination microscopy (SIM) has become a widely used tool for insight into biomedical challenges due to its rapid, long-term, and super-resolution (SR) imaging. However, artifacts that often appear in SIM images have long brought into question its fidelity, and might cause misinterpretation of biological structures. We present HiFi-SIM, a high-fidelity SIM reconstruction algorithm, by engineering the effective point spread function (PSF) into an ideal form. HiFi-SIM can effectively reduce commonly seen artifacts without loss of fine structures and improve the axial sectioning for samples with strong background. In particular, HiFi-SIM is not sensitive to the commonly used PSF and reconstruction parameters; hence, it lowers the requirements for dedicated PSF calibration and complicated parameter adjustment, thus promoting SIM as a daily imaging tool.

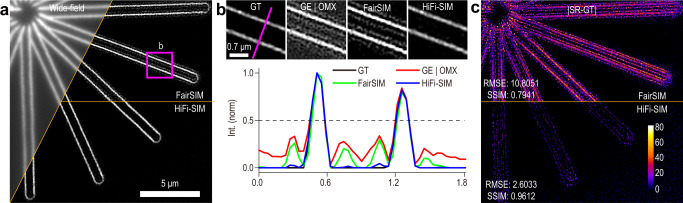

## Introduction

Super-resolution structured illumination microscopy (SR-SIM), which breaks the optical diffraction barrier, offers an unprecedented opportunity for investigating biological structures at a ~100 nm scale^1,2^. Despite the relatively modest spatial resolution improvement compared to other SR techniques^3,4^, SR-SIM is recognized as one of the most promising tools for studying the dynamics of subcellular structures in live cells due to its high photon efficiency, insignificant photon damage, and bleaching, as well as compatibility with most fluorescent labeling protocols^5–10^. For example, moving vesicles in the endoplasmic reticulum (ER) were imaged by Hessian-SIM with a spatiotemporal resolution of 88 nm and 188 Hz^8^. The cortical ER (cER) network near the basal cell cortex were imaged by GI-SIM at 97 nm resolution and 266 Hz frame-rate over thousands of time points^9^. Recently, the GPU-accelerated technology realized multi-color instant SR-SIM at video frame-rate, with a delay of less than 250 ms between acquisition and reconstruction^11^.

Despite these advances, the fidelity and quantification of SR-SIM are often challenged^12,13^ because the final SR images heavily rely on post-processing algorithms that are prone to reconstruction artifacts^8,14,15^. To acquire SIM images with minimal artifacts, dedicated practical guidelines were recommended for instrument refinement^16^, data acquisition^17,18^, and sample preparation^18,19^. Several studies have been conducted on reconstruction algorithms, including accurate illumination parameter estimation^20–24^, iterative deconvolution^8,25,26^, and fine tuning of reconstruction parameters^27^. Despite all these efforts, artifacts that limit the implementation of SIM as a daily imaging tool still frequently appear in SIM images. In particular, new structures discovered using SR-SIM need to be interpreted with special care to avoid misinterpretation. Recently, deep learning has shown great potential for SR-SIM reconstruction^28,29^, but the results are closely related to SR images obtained via other methods for training neural network, thus the fidelity is still questioned.

In this study, we demonstrate a novel SIM algorithm based on PSF engineering termed as High-Fidelity SIM reconstruction algorithm (HiFi-SIM) that can reconstruct SR images with minimal artifacts and optimal optical sectioning (OS). By combining the normalized cross-correlation method with a spectrum notch, HiFi-SIM can automatically estimate reconstruction parameters from the majority of raw data. With a two-step spectrum optimization, HiFi-SIM can effectively suppress commonly seen artifacts and remove residual background fluorescence without losing fine and weak structures. HiFi-SIM uses a theoretical PSF and has a few user-defined parameters, avoiding stringent PSF calibration and complex parameter adjustments. We tested HiFi-SIM on images of standard samples of representative structures and biological samples of various qualities. The results were compared with that of several commercial setups and open-source algorithms, and HiFi-SIM demonstrated a superior performance among all the other methods.

## Results

### HiFi-SIM principle

**Fig. 1:**
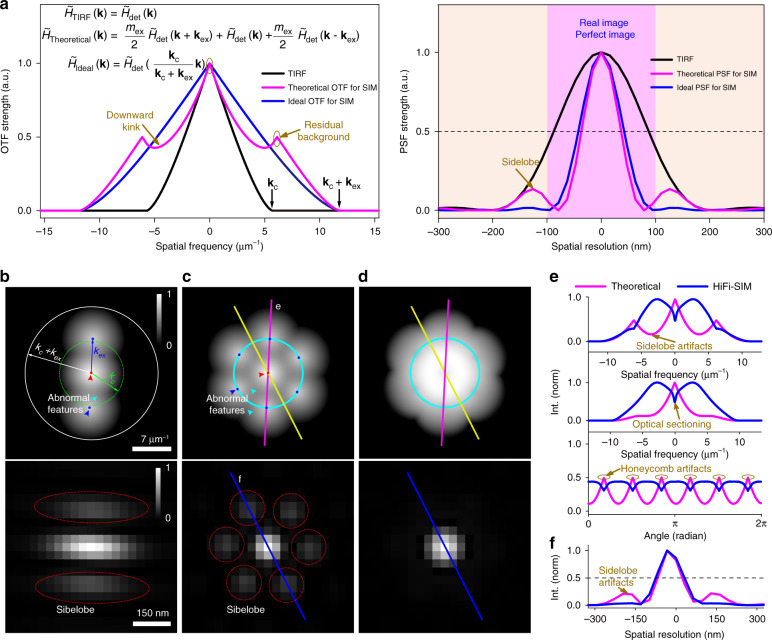
Underlying principle of PSF engineering. **a** Theoretical OTFs and corresponding PSFs for wide-field fluorescence microscopy (total internal reflection fluorescence microscopy, TIRF) and directly combined SR-SIM, and the ideal OTF and corresponding PSF for SR-SIM. The downward kinks in the OTF of directly combined SR-SIM result in sidelobes in PSF, while background fluorescence signals located at the upward peaks of the OTF cause honeycomb artifacts and limit the OS capability. $\tilde H_{{\mathrm{det}}}\left( {\mathbf{k}} \right)$ represents the actual OTF for wide-field imaging. **k**_*c*_ represents the cut-off frequency of wide-field imaging. ***m***_ex_ and **k**_ex_ represent the modulation depth and spatial frequency of the excitation pattern, respectively. **b** 2D OTF and corresponding PSF of directly combined SR-SIM with excitation pattern in only one orientation. **c** 2D OTF and corresponding PSF of directly combined SR-SIM with excitation patterns in three orientations. Green and white circles represent the diffraction limited boundaries of the wide-field and SR-SIM, respectively; blue spots represent the spatial frequencies of the excitation pattern at different orientations; cyan circles represent the circular cross-section with a radius equal to the spatial frequency of the excitation patterns. **d** Equivalent OTF and corresponding PSF after optimizing the directly combined OTF by HiFi-SIM. **e** Intensity profiles along the magenta, yellow, and cyan lines in **c** and **d**. **f** Intensity profiles along the blue lines in **c** and **d**. Gamma value: 0.3 for OTFs in **b**–**d**

To date, the reconstruction algorithms used by most commercial SIM setups and successful open-source packages, such as SIMToolbox^30^, fairSIM^31^, and OpenSIM^32^, were based on the Wiener deconvolution procedure established by Heintzmann^33^ and Gustafsson^34^ (hereinafter referred to as “Wiener-SIM”). Some iterative deconvolution algorithms, such as total variation (TV)-SIM^8,25^ and Hessian-SIM^8^, are also based on the results of Wiener-SIM. Reconstruction of Wiener-SIM is conducted by the recombination of different spectrum components in Fourier domain, which inevitably leads to an equivalent optical transfer function (OTF) with non-smooth shape^4,7^ ($\tilde H_{{\mathrm{Theoretical}}}\left( {\mathbf{k}} \right)$ in Fig. 1a). By inverse Fourier transformation, the downward kinks in the OTF are transformed into sidelobes in the PSF. The abnormal features of OTF could be corrected by Wiener deconvolution, but this requires that the OTF used and estimated reconstruction parameters accurately match the actual imaging conditions^8,15,18,27^; otherwise, the final SR images often exhibit reconstruction artifacts, such as sidelobe artifacts. Moreover, the residual background signals are usually concentrated at the raised peaks of $\tilde H_{{\mathrm{Theoretical}}}\left( {\mathbf{k}} \right)$, causing periodic honeycomb artifacts^7,12,18^ and limiting the OS capability^35–38^. This defect is usually remedied by OTF attenuation^20,27,31,36^, but this solution cannot achieve a good balance between rejecting background fluorescence and protecting fine and weak structures. For a more detailed discussion of the thorny issues in Wiener-SIM reconstruction, see Supplementary Note 1.

The ideal OTF for SR-SIM ($\tilde H_{{\mathrm{ideal}}}\left( {\mathbf{k}} \right)$) should be a similar form to that of wide-field OTF ($\tilde H_{{\mathrm{det}}}\left( {\mathbf{k}} \right)$) but with extended cut-off frequency (Fig. 1a). The idea is to design an optimization function $\tilde W({\mathbf{k}})$ applied to $\tilde H_{{\mathrm{Theoretical}}}\left( {\mathbf{k}} \right)$ to make it as close as possible to the ideal form

1 $$\tilde H_{{\mathrm{Theoretical}}}\left( {\mathbf{k}} \right) \cdot \tilde W({\mathbf{k}}) \cong \tilde H_{{\mathrm{ideal}}}\left( {\mathbf{k}} \right) = \tilde H_{{\mathrm{det}}}\left( {\frac{{{\mathbf{k}}_c}}{{{{\mathbf{k}}_c + {\mathbf{k}}_{{\mathrm{ex}}}} }}{\mathbf{k}}} \right)$$

where **k**_*c*_ is the cut-off frequency of wide-field imaging, and **k**_ex_ is the spatial frequency of the excitation pattern. With Eq. (1), at least in principle, SR-SIM could achieve perfect imaging without sidelobe (blue lines in Fig. 1a).

Inspired by the above, we developed a high-fidelity SIM reconstruction algorithm by engineering the equivalent PSF of SR-SIM into an ideal form, called “HiFi-SIM”. The flowchart of HiFi-SIM is shown in Fig. S1, which contains three main steps. In preprocessing, the raw data was deconvoluted^26^ using a theoretical OTF. In reconstruction parameter estimation, an improved normalized cross-correlation method was used to estimate the pattern wave vectors. In short, a notch filter was applied to the normalized cross-correlation calculation^8,23^, which can effectively suppress contributions from low-frequency signal and local periodic structures to the cross-correlation map, such that the peaks corresponding to the wave vectors protruded enough to be automatically determined from most of raw data, including low SNR data, TIRF-SIM data, and even data with obvious periodic structures (Fig. S2). Thus, a pre-set mask or hand-correction was not required as before. Once the reconstruction parameters were correctly determined, the 0- and ±1-order spectrum components were separated, shifted, and combined to yield a directly combined spectrum $\tilde S_{{\mathrm{directly - combined}}}({\mathbf{k}})$ (Eq. (9) in Supplementary Note 1), using a method similar to fairSIM. After that, HiFi-SIM performs a two-step spectrum optimization on $\tilde S_{{\mathrm{directly - combined}}}({\mathbf{k}})$.

The first step is to construct an initial optimization function $\tilde W_1\left( {\mathbf{k}} \right)$ to correct $\tilde H_{{\mathrm{Theoretical}}}\left( {\mathbf{k}} \right)$ to be close to $\tilde H_{{\mathrm{ideal}}}\left( {\mathbf{k}} \right)$, as in Eqs. (20, 21) of Supplementary Note 2. A weight function $\tilde g_1\left( {\mathbf{k}} \right)$ is used in $\tilde W_1\left( {\mathbf{k}} \right)$ to modulate all the raised peaks into inverted peaks (Fig. 1b–e) to remove the residual background signals. With the initial optimization, the abnormal features in $\tilde S_{{\mathrm{directly - combined}}}({\mathbf{k}})$ can be well corrected, so the typical artifacts and residual background can be effectively eliminated (Fig. S3a, b, d, e). Following $\tilde W_1\left( {\mathbf{k}} \right)$ optimization, another deconvolution function $\tilde W_2\left( {\mathbf{k}} \right)$ based on $\tilde H_{{\mathrm{Theoretical}}}\left( {\mathbf{k}} \right)$ is developed to recover the high-frequency signals suppressed by the optimized OTF, as in Eqs. (12, 22, 23) of Supplementary Notes1 and 2. Like the challenge of applying OTF attenuation in Wiener-SIM (Supplementary Note 1), the modulation of $\tilde g_1\left( {\mathbf{k}} \right)$ may impair the fidelity of the initial optimized spectrum to some extent, resulting in the loss of some fine and weak signals (S3g, h). To this end, another weight function $\tilde g_2\left( {\mathbf{k}} \right)$ is used in $\tilde W_2\left( {\mathbf{k}} \right)$ to actively compensate the attenuated regions of the spectrum optimized by $\tilde W_1\left( {\mathbf{k}} \right)$. Thus $\tilde W_2\left( {\mathbf{k}} \right)$ not only improves the spatial resolution, but also recovers the real sample signals lost during $\tilde W_1\left( {\mathbf{k}} \right)$ optimization (Fig. S3h, i).

With the two-step spectrum optimization, the equivalent OTF displayed features with smooth axial distributions at all directions, relative uniform distribution along circumference, and a collapse downward at the center, as shown in Fig. 1d, e. Therefore, a high-fidelity SR-SIM was realized with effectively suppressed artifacts and fully preserved fine structures. Moreover, the two-step spectrum optimization overcomes the long-term challenge of OTF mismatch for Wiener-SIM-based algorithms (Supplementary Note 1), as shown in Figs. S4 and S5. Thus, a theoretical OTF is good enough to generate SR images without observable artifacts, hence it is used in HiFi-SIM by default. See Supplementary Notes2 and 3 for a more detailed discussion of the principle and implementation of HiFi-SIM.

### Fidelity characterization with standard samples

**Fig. 2:**
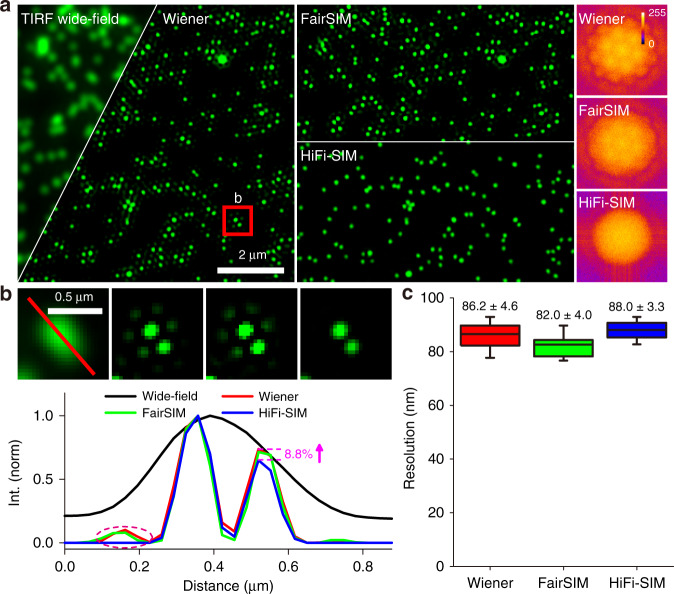
Experimental characterization of the correlation between artifacts and abnormal features of reconstructed spectrum. **a** SR images of 100 nm fluorescent microspheres collected from our home-built TIRF-SIM setup were reconstructed using the traditional Wiener-SIM we implemented, fairSIM, and HiFi-SIM, and the corresponding reconstructed spectrum is shown on the right. Note that the reconstructed spectrum of traditional Wiener-SIM and fairSIM both show obvious patchy features, and the real microspheres in the SR images contain obvious sidelobe artifacts. **b** Magnified images of the red-box region in **a**, and the line profiles along the microspheres in **b** show diminishing sidelobe artifacts in HiFi-SIM. **c** Full-width half-maxima (FWHMs) of the fluorescence profiles of 10 microspheres in the reconstruction images of traditional Wiener-SIM, fairSIM, and HiFi-SIM

Three representative structures, including microspheres, lines, and rings, were employed as standard samples to quantitatively characterize the fidelity of HiFi-SIM. Fluorescent microspheres of 100 nm diameter were imaged by a home-built laser-interference SIM (Fig. S6) and reconstructed with the traditional Wiener-SIM we implemented, fairSIM, and HiFi-SIM (Fig. 2). Using a theoretical OTF (Eq. (14) in Supplementary Note 1: “dampening” factor = 1), the spectrum reconstructed by traditional Wiener-SIM displayed patchy features (Fig. 2a), which led to obvious sidelobe artifacts with an intensity about 10% of the real microspheres (Fig. 2b). With an active compensation for the theoretical OTF (“dampening” factor = 0.3)^27,31^, the patchy features became weaker but still existed in the spectrum of fairSIM; hence, the SR image showed similar sidelobe artifacts. By contrast, the spectrum reconstructed by HiFi-SIM using the same OTF as in traditional Wiener-SIM showed homogenous features, yielding clean microspheres. As shown in Fig. 2b, the sidelobe artifacts may be superimposed on the adjacent actual microsphere, increasing its strength by about 8.8%. Because the energy distribution in the PSF of HiFi-SIM is mainly concentrated in the main lobe (Fig. 1a, f), the average FWHM of individual microspheres was slightly increased by about 6 nm (Fig. 2c), but the detailed features were easier to identify due to fewer artifacts.

The actual OTF of the system could be determined from the corresponding wide-field images^32^. With the measured OTF, the patchy features in the spectrum reconstructed by traditional Wiener-SIM, re-Wiener, TV-SIM, and Hessian-SIM were reduced but sidelobe artifacts with a strength of approximately 3–5% still existed around many microspheres (Fig. S7), indicating an inherent problem in the combined spectrum. Instead, HiFi-SIM is not sensitive to the OTF used, yielding clean microspheres without sidelobe artifacts (Figs. 2a, b and S4c–e). This made the results of HiFi-SIM more trustworthy, especially for a case when an accurately calibrated OTF is not available. As a similar spherical structure in biology, caveolae in live U2OS cells were imaged by Nikon N-SIM (Fig. S8a) and reconstructed by NIS-elements using the calibrated OTF. The result showed similar snowflake-like sidelobe artifacts, which are difficult to distinguish from some real but weak caveolae (Fig. S8b, e). Utilizing HiFi-SIM, the quality and fidelity of SR image of the caveolae was considerably improved (Fig. S8c, f).

**Fig. 3:**
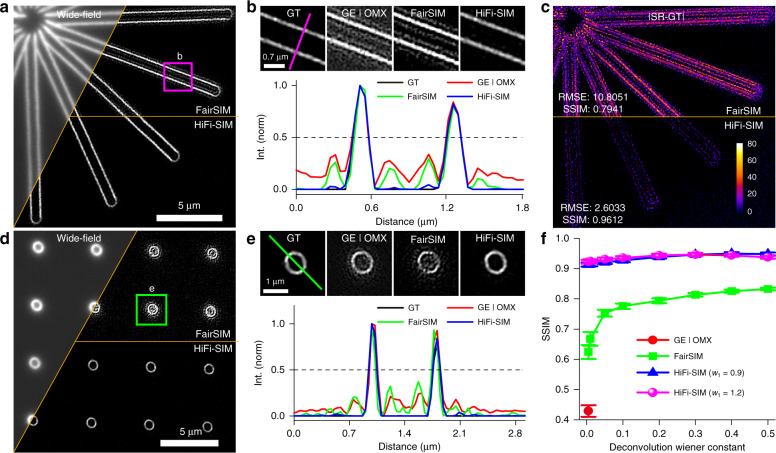
Quantitative characterization of the fidelity of HiFi-SIM reconstruction. **a** “Start” patterns in Argolight slide were imaged by DeltaVision OMX with 5 ms exposure. The equivalent wide-field image is shown in the upper-left, and the SR images of fairSIM and HiFi-SIM are presented in the upper-right and lower-right corners, respectively. **b** Magnified SR images reconstructed from the magenta-box region in **a** using GE SoftWoRx, fairSIM, and HiFi-SIM. The ground-truth (GT) is shown on left, which is reconstructed by HiFi-SIM from the same region of high-quality data with 50 ms exposure. Intensity profiles along the magenta line in **b** confirm that HiFi-SIM eliminates almost all sidelobe artifacts around the real lines. **c** Error-map of the SIM images to the ground-truth model. RMSE and SSIM values are shown in the lower-left corner. **d**, **e** Comparison of the SR images for “ring” patterns in Argolight slide with 10 ms exposure time, reconstructed by fairSIM, HiFi-SIM, and SoftWoRx. **f** SSIM plot of reconstructed SR results vs. the ground-truth model, with different reconstruction parameters

Figure 3 shows the results of line and ring structures from an Argolight standard sample imaged with GE DeltaVision OMX. Artificial lines parallel to the real structures with an intensity of approximately 10–30% of the actual structures appeared in the SR images obtained by GE SoftWoRx using the calibrated OTF and fairSIM using a theoretical OTF (Fig. 3a, b). Similar artificial structures also appeared in the reconstruction results of TV-SIM and Hessian-SIM (Fig. S9a, c). With HiFi-SIM, these sidelobe structures were effectively eliminated. The root mean square error (RMSE) of the error-map to the ground-truth (GT) model (Fig. S10) was decreased approximately four times by HiFi-SIM than that by fairSIM (Fig. 3c). For the ring structures, artifacts in the center are usually more obvious than those on the outside, because the sidelobes in the inside overlap (Figs. 3d, e and S9b, d). As expected, HiFi-SIM effectively eliminated these artifacts, yielding clean hollow rings. The calculated structural similarity remained above 91.3% to the GT model, even with different reconstruction parameters (*w*_1_ and *w*_2_), as shown in Fig. 3f. This suggests that SR images by HiFi-SIM are not sensitive to the reconstruction parameters. Thus, routine adjustment is not required for most cases, which considerably facilitates the application of SIM as a daily imaging tool.

### Artifacts suppression by HiFi-SIM for high-quality data

**Fig. 4:**
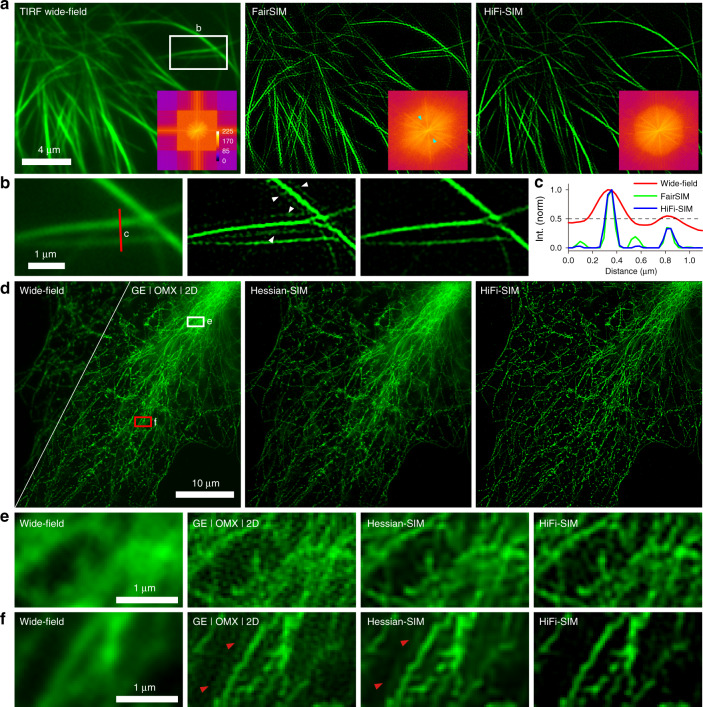
Performance of HiFi-SIM on reconstructing raw data with high-quality. **a** TIRF wide-field image of microtubules in live COS-7 cells is shown in the left, and the SR images of fairSIM and HiFi-SIM are presented in the middle and right, respectively. Lower-right corner shows corresponding reconstructed spectrum. **b** Magnified images of the white-box region in **a**. **c** Intensity profiles along the red line in **b**. **d** Equivalent wide-field image of microtubules in fixed COS-7 cells is shown in the left triangle, and the SR images were reconstructed using GE SoftWoRx, Hessian-SIM, and HiFi-SIM, respectively. **e**, **f** Magnified images of the wide-field equivalent in the white-box and red-box regions in **d** are shown in the left, and the corresponding SR images were reconstructed by SoftWoRx, Hessian-SIM, and HiFi-SIM, respectively. Here, Hessian-SIM uses the same OTF as used in fairSIM (Fig. S11), and uses the built-in notch filters to suppress pattern artifacts (*a*_0_ = 0.05, *v* = 1.2)

Successful SIM imaging generally requires structured illumination pattern with high modulation depth, which is best obtained in TIRF-SIM mode. We imaged microtubules in live COS-7 cells using TIRF-SIM mode of the home-built setup, and the raw data were reconstructed with fairSIM and HiFi-SIM (Fig. 4a). High modulation depth (greater than 0.5) enabled both algorithms to reconstruct high-quality SR images. However, with fairSIM, sidelobe artifacts, with an intensity of approximately 10% of the actual microtubules, were present in certain regions, and an overlap with two sidelobes from nearby microtubules created a structure with doubled intensity, which could very likely be interpreted as a real structure (Fig. 4b, c). With HiFi-SIM, the abnormal spectrum could be effectively corrected, thereby leaving no observable sidelobe artifacts (Fig. 4a–c).

Microtubules in a fixed COS-7 cells were further imaged by GE DeltaVision OMX under conventional 2D-SIM mode (incident beam angle smaller than the critical angle of TIRF). The raw data have high modulation depths (0.62, 0.76, and 0.82 in three orientations) but also have a large dynamic range due to the strong background at the right upper corner. SR images reconstructed by GE SoftWoRx, fairSIM, Richardson-Lucy deconvolution (RL)-SIM^26^, TV-SIM, and Hessian-SIM contain obvious residual background and sidelobe artifacts, especially the strong background area (Figs. 4d–f and S11). In particular, with TV- and Hessian-denoising, the fibers seem to be more continuous, but the sidelobe artifacts cannot be eliminated (Figs. 4f, S11c and S12). By contrast, HiFi-SIM effectively removed the residual background and typical artifacts, yielding SR images with higher fidelity and contrast (Figs. 4d–f and S12). Additionally, HiFi-SIM was applied to many high-quality external datasets. In all tested cases, HiFi-SIM yielded SR images with better quality (Fig. S13).

### Reconstruction of suboptimal data by HiFi-SIM

**Fig. 5:**
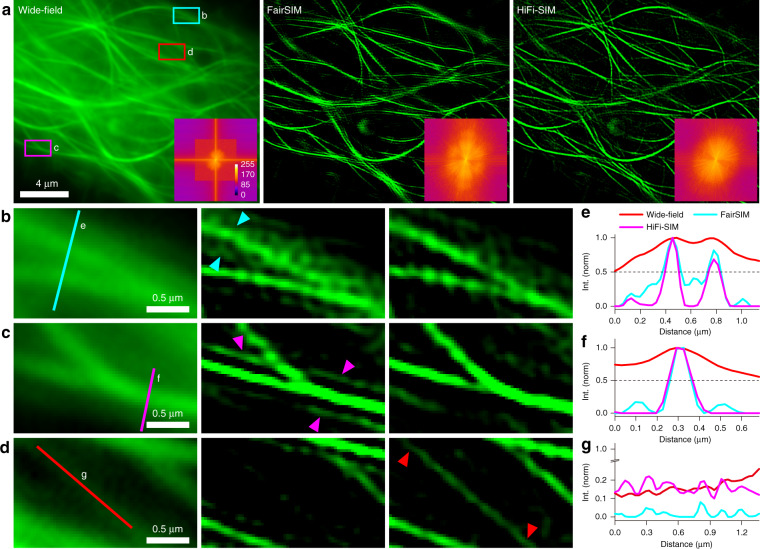
Performance of HiFi-SIM on reconstructing raw data with low modulation depth and strong background. **a** Equivalent wide-field image of microtubules in live U2OS cells with strong background fluorescence is shown in the left, and the SR images reconstructed using fairSIM and HiFi-SIM. Lower-right corner shows corresponding reconstructed spectrum. **b**–**d** Magnified images of the red-box, cyan-box, and magenta-box regions in **a**. **e**–**g** Intensity profiles along the red, cyan, and magenta lines in **b**–**d**, respectively

Generally, suboptimal raw data (modulation depth lower than 0.5) is recommended to be treated with special care or abandoned due to the higher risk of artifacts. To mimic suboptimal SIM imaging, microtubules in live U2OS cells were imaged in conventional SIM mode (Fig. 5a). Particularly, the modulation depths were intentionally adjusted to be different (0.43, 0.36, and 0.21 in three orientations) by controlling the beam polarization^16^. To reconstruct an acceptable SR image with fairSIM, OTF attenuation strength was increased to reduce artifacts and residual out-of-focus background. This caused the loss of some tiny and weak structures, but serious honeycomb and sidelobe artifacts still exist (Fig. 5b–d). In comparison, HiFi-SIM reconstructed SR image with little honeycomb and sidelobe artifacts, and the lost tiny structures were preserved (Fig. 5a–g). This demonstrates that HiFi-SIM can better balance the trade-off between rejecting out-of-focus signal and preserving fine and weak structures (Figs. S12 and S14).

SR images of raw data with a strong background also often suffer from hammerstroke artifacts derived from non-modulated background or high-frequency noise^18^. Further Hessian denoising can effectively suppress the hammerstroke artifacts, but may enhance the non-continuous segments around the real structures into continuous sidelobe artifacts^10,11^ (Figs. S15 and S16). By using HiFi-SIM, the reconstructed images have less hammerstroke and sidelobe artifacts.

### Optical sectioning by HiFi-SIM

In biological application, there is demand to quickly capture 3D cell structures from volume stack of 2D-SIM imaging. But it is generally regarded that 2D-SIM lack optical sectioning capability due to the missing-cone of 3D OTF^1,2,4,39,40^. In HiFi-SIM, the two-step spectrum optimization reshapes the equivalent OTF to be concave downward at the center (Fig. 1d, e). In the corresponding 3D OTF, the missing cone will partly be filled so that OS capability could be achieved. To demonstrate this, microfilaments in fixed U2OS cells were imaged by DeltaVision OMX. Reconstructed results by HiFi-SIM showed higher contrast and fewer artifacts than the results by GE SoftWoRx (Fig. S17). Moreover, HiFi-SIM has been extended to reconstruct single-layer 3D-SIM datasets (Supplementary Note 2), and its reconstruction quality was comparable to the quality of the same layer in full 3D-SIM reconstruction (Figs. S18 and S19), although there is no 2-fold axial resolution improvement.

**Fig. 6:**
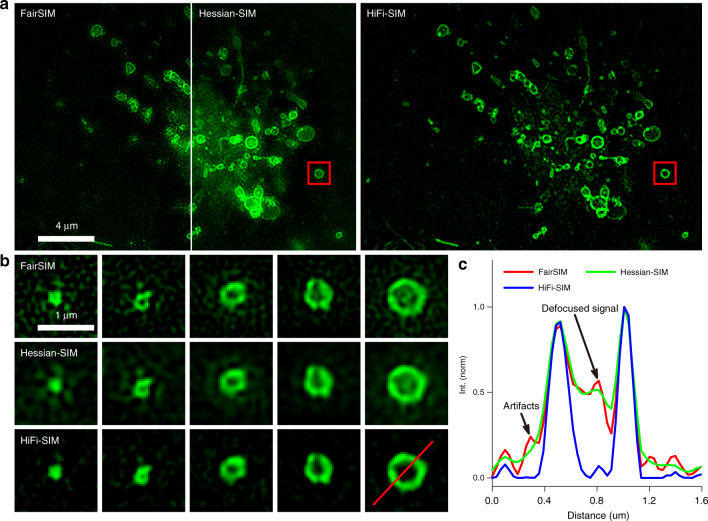
Imaging of vesicle in live U2OS cells. **a** Comparison of SR images reconstructed by fairSIM (left), Hessian-SIM (middle), and HiFi-SIM (right). **b** Magnified SR images of vesicles with different sizes using fairSIM (top), Hessian-SIM (middle), and HiFi-SIM (bottom). Note that vesicles with larger sizes reconstructed by fairSIM have obvious defocused background artifacts in the center and outside. Further performing the Hessian denoising procedure on the results of fairSIM can suppress the artifacts to a certain extent, but cause the center of the vesicles to be blurred. **c** Intensity profiles along the red line in **b**

We further imaged moving vesicles in live U2OS cells using conventional 2D-SIM mode of the home-built setup. Membrane proteins were labeled with CD63-EGFP, such that each vesicle represents a spherical structure. In 2D-SIM imaging, these vesicles appear as microspheres or hollow rings depending on their size, i.e., whether they are smaller or larger than the SIM resolution. However, most hollow vesicles reconstructed by fairSIM and Hessian-SIM contain serious artifacts and residual defocused background both in the center and outside (Fig. 6), similar to the 2D-SIM images of the standard rings (Fig. 3d, e). Instead, HiFi-SIM can reconstruct vesicles of different sizes into microspheres or central hollow rings with minimal artifacts, which is consistent with the result of full 3D-SIM (Fig. S19).

## Discussion

We developed a high-fidelity SIM algorithm to reconstruct high-quality SR images. Previously, to ensure high-quality SR-SIM images, careful experiment design, dedicated system OTF calibration, and fine-tuning of multiple user-defined parameters were typically required. This set high entry barriers for ordinary SIM users. The proposed HiFi-SIM is more immune to reconstruction artifacts related to OTF mismatch than most Wiener-SIM-based algorithms (Figs. S4 and S5). Thus, a theoretical OTF is used by default in HiFi-SIM, avoiding complicated OTF calibration. For high-quality data with high modulation depth (greater than 0.5), usually only one parameter (*attStrength*) needs to be adjusted for optimal OS performance (Fig. S20). For suboptimal data with low modulation depth (between 0.1 and 0.5) or strong background fluorescence, adjusting only two additional parameters (*ApoFWHN* or *β*) accordingly usually ensures good quality SR images. An easy-to-implement guideline is provided in Supplementary Note 3.

Raw data with low SNR is notoriously difficult to reconstruct acceptable SR images. In traditional Wiener-SIM, the Wiener deconvolution amplifies the unwanted high-frequency noise signal^8,25^ (Supplementary Note 1). Hence, random discontinuous artifacts frequently appear in SIM images of low SNR raw data. In HiFi-SIM, appropriately reducing *ApoFWHN* can suppress random discontinuous artifacts related to high-frequency noise (Fig. S21). Intentionally enhancing the low- and medium-frequency signals by appropriately increasing *β* can also improve the reconstruction quality of low SNR data (Figs. S22 and S23; Supplementary Note 3). Both TV-SIM and Hessian-SIM can also suppress the random discontinuous artifacts excellently, but they are not able to eliminate the sidelobe artifacts and residual background (Figs. 4e, f, S9, S11, and S12). Moreover, in some cases, Hessian denoising may cause discontinuous segments to be enhanced into continuous sidelobes (Figs. S15 and S16), which may increase the risk of artifacts. For raw data with extremely low SNR or thick samples with strong background, Hessian-SIM may perform better than HiFi-SIM to reduce hammerstroke artifacts. In this case, further Hessian denoising could be performed on the results of HiFi-SIM to yield better SR images.

Recently, Hoffman et al. developed Tiled-SIM which divides the raw data into overlapping tiled subsets and reconstructs each subset with independent parameters^13^. As HiFi-SIM is not sensitive to reconstruction parameters, this tiled method is not necessary in most cases. For raw data with large aberrations or high dynamic range in the entire field of view, applying HiFi-SIM to reconstruct each tiled subset can further improve the quality of Tiled-SIM (Fig. S24e, f). Results of HiFi-SIM could be used as input to train the neural network for deep-learning based methods to improve their fidelity^28,29^. The principle of spectrum optimization in HiFi-SIM could also be applied to reconstruct images of non-linear SIM^7,12,41^ and lattice light-sheet microscopy^42^, which are more prone to abnormal spectrum and artifacts. In the future, GPU acceleration^11^ could be conducted for HiFi-SIM to speed up reconstruction.

In summary, HiFi-SIM provides an easy-to-use SIM reconstruction approach based on PSF engineering, yielding high-fidelity SR images from varied quality raw data. Compared with other technologies, HiFi-SIM can lower the heightened standards for experimental implementation and post-processing reconstruction, hence improving the accessibility of SR-SIM for ordinary users.

## Materials and methods

### Standard fluorescent sample preparation

Fluorescent microspheres of 100-nm-diameter and commercial Argo-SIM slide were employed as standard samples to quantitatively evaluate the fidelity of reconstruction algorithms. Carboxylate-modified microspheres (0.1 µm, yellow-green fluorescent 505/515, F8803) were purchased from Thermo Fisher Scientific (MA, USA) and diluted 100 times before use. Commercially available coverslips (~150 μm thick) with 24 × 60 mm (Cellvis, USA) were carefully cleaned using the procedure in ref. ^11^. A silicone sheet (GBL665201-25EA, Sigma-Aldrich, USA) with a 9-mm diameter well was attached to the coverslip. The fluorescent solution was dispensed onto the coverslip and imaged in PBS buffer. The “Star” and “2D matrix of rings” patterns in Argo-SIM were used as typical “line” and “ring” structures.

### Cell culture and labeling

COS-7 and U2OS cells were obtained from the Cell Bank of the Chinese Academy of Sciences (Shanghai, China) and cultured in an incubator at 37 °C and 5% CO_2_. The COS-7 cells were cultivated in a DMEM medium (Thermo Fisher Scientific, USA) supplemented with 1% penicillin G, streptomycin (Sangon Biotech, China), and 10% fetal bovine serum (Thermo Fisher Scientific, USA). The U2OS cells were cultivated in McCoy’s 5A medium, modified (Thermo Fisher Scientific, USA), supplemented with 1% penicillin G, streptomycin (Sangon Biotech, China), and 10% fetal bovine serum (Thermo Fisher Scientific, USA).

Cells were transiently transfected using Lipofectamine 2000 (Thermo Fisher Scientific, USA) as per manufacturer’s protocol. The mEmerald-Tubulin-N-18 vector (plasmid #54293, Addgene, USA), mEGFP-Lifeact vector (plasmid #54610, Addgene, USA), mEmerald-Caveolin vector (plasmid #54025, Addgene, USA), and mEmerald-ER-3 vector (plasmid #54082, Addgene, USA) were used to label the microtubule, microfilament, caveolae, and endoplasmic reticulum, respectively. Cell vesicles were labeled by the CD63-EGFP vector, which was constructed by inserting Homo sapiens CD63 cDNAs into pEGFP-n1 vector (Clontech, USA). Twenty-four hours after transfection, the cells were detached using trypsin-EDTA (Thermo Fisher Scientific, USA), seeded onto poly-L-lysine-coated 35-mm glass-bottom dishes (Cellvis, USA), and cultured in an incubator at 37 °C and 5% CO_2_ for an additional 24 h before the experiments.

For live cell imaging, the complete medium was replaced by HBSS solution (Thermo Fisher Scientific, USA) containing Ca^2+^ and Mg^2+^ but no phenol red. For fixed cell imaging, the complete medium was removed and cells were fixed with 4% paraformaldehyde for 10 min at room temperature. After fixation, cells were washed twice by PBS buffer. Both live and fixed cells were imaged in PBS buffer.

### SIM imaging

SIM experiments were performed using commercial SIM microscopes, namely DeltaVision OMX SR (GE Healthcare in Issaquah, Washington, USA) and N-SIM S (Nikon Corporation, Tokyo, Japan), as well as a custom-built two-beam interference SIM microscope (Fig. S6). The custom-built SIM was constructed around a commercial inverted fluorescence microscope (IX83, Olympus Life Science, Japan) with a TIRF-oil-immersion objective (UAPON 100×, NA = 1.49, Olympus Life Science, Japan). A 488-nm, 500-mW semiconductor laser (Genesis MX488-500 STM, Coherent, USA) was used for excitation, a quad-band total internal reflection (TIRF) filter block (TRF89902-EM, Chroma, USA) was employed for imaging, and a sCMOS camera (ORCA-Flash 4.0 V2, Hamamatsu, Japan) was used as the detector. To generate illumination patterns with different periods, a ferroelectric liquid-crystal spatial light modulator (SLM, SXGA-3DM, Fourth Dimension Displays, UK) was employed as the grating.

Microspheres with 100 nm diameter, microtubules, and microfilaments in live COS-7 cells were imaged using the custom-built setup in TIRF-SIM mode. Microtubules and vesicles in live U2OS cells, and endoplasmic reticulum in fixed U2OS cells were imaged in the setup under conventional SIM mode with incident beam angle smaller than the critical angle of TIRF. Reconstruction parameters: for microspheres data, emission wavelength (*λ*_em_) = 515 nm while that for microtubule, microfilaments, vesicles, and endoplasmic reticulum data was 525 nm, and the single pixel size of the detector was calibrated to 65 nm/pixel.

The “2D matrix of rings” and “Star” patterns in Argo-SIM, microtubules in fixed COS-7 cells, and microfilaments in fixed U2OS cells were imaged on the DeltaVision OMX SR with the parameters: NA = 1.42 (oil immersed), excitation wavelength (*λ*_ex_) = 488 nm, emission wavelength (*λ*_em_) = 527 nm, and the single pixel size of the detector was calibrated to 78.6 nm/pixel. In addition, caveolae in live U2OS cells were imaged on the N-SIM S with the parameters: NA = 1.49 (oil immersed), excitation wavelength (*λ*_ex_) = 488 nm, emission wavelength (*λ*_em_) = 525 nm, and the single pixel size of the detector was calibrated to 60 nm/pixel.

### Image reconstruction

Commercial SIM reconstruction software packages, including GE SoftWoRx and Nikon NIS-Elements, and open source packages, including fairSIM, SIMToolbox, and iterative deconvolution procedures in ref. ^8^, were used for comparative SR image reconstruction. Images labeled ‘GE | OMX’ were reconstructed with SoftWoRx, and the Wiener constants were 0.005 by default. Images labeled ‘Nikon | N-SIM | 2D’ were reconstructed with NIS-Elements. Images labeled ‘FairSIM’ and ‘RL-SIM’ were reconstructed with the Wiener-SIM and RL-SIM in fairSIM, respectively. The adopted OTFs were theoretical approximate OTFs (‘dampening’ factor = 0.3), and Wiener constants defaulted to 0.1. Images labeled ‘SIMToolbox | Wiener’ and ‘Map-SIM’ were reconstructed with the Wiener-SIM and Maximum a posteriori probability SIM (Map-SIM) in SIMToolbox. Images labeled ‘re-Wiener’, ‘TV-SIM’, and ‘Hessian-SIM’ were reconstructed with the Wiener-SIM, TV and Hessian denoising procedures in ref. ^8^. As a comparison with HiFi-SIM, we have also implemented a traditional Wiener-SIM. In the implementation, raw data preprocessing, and reconstruction parameter estimation use the same methods as in HiFi-SIM, whereas the recombination of spectrum components follows the traditional Wiener deconvolution procedure (Supplementary Note 1). Images labeled ‘Wiener’ were reconstructed with the traditional Wiener-SIM implemented by us.

### Quantification of the fidelity of SR images

To quantitatively evaluate the fidelity of SR images reconstructed by HiFi-SIM, two typical patterns (rings and lines) of known real structures in the Argo-SIM slide were employed as standard samples for 2D-SIM imaging (Figs. 3 and S9). Because structures of the samples were known (rings and lines), raw data with high modulation and high SNR were collected with the exposure time of 50 ms, and SR images with minimal artifacts were reconstructed thereafter. The residual noise in the SR image was eliminated by setting thresholds, and clean SR images were obtained as the ground-truth models (Fig. S10). Error maps and corresponding RMSE values, between the SR and ground-truth images, were displayed to evaluate the fidelity of reconstruction algorithms (Fig. 3c). Furthermore, the structure similarity index measure (SSIM) was used to quantitatively evaluate the fidelity of SR images, defined as

$${\mathrm{SSIM}}(I^{{\mathrm{SR}}},I^{{\mathrm{GT}}}) = \frac{{(2\mu _{{\mathrm{SR}}}\mu _{{\mathrm{GT}}} + C_1)(2\sigma _{{\mathrm{SR}},{\mathrm{GT}}} + C_2)}}{{(\mu _{{\mathrm{SR}}}^2 + \mu _{{\mathrm{GT}}}^2 + C_1)(\sigma _{{\mathrm{SR}}}^2 + \sigma _{{\mathrm{GT}}}^2 + C_2)}}$$

where *μ*_SR_ and *μ*_GT_ are the mean values of images *I*^SR^ and *I*^GT^, respectively; *σ*_SR_ and *σ*_GT_ are the standard deviations of *I*^SR^ and *I*^GT^, respectively; and *σ*_SR*,*GT_ is the cross-variance between images *I*^SR^ and *I*^GT^. *C*_1_ and *C*_2_ are used to avoid division by a small denominator and set as *C*_1_ = 0.05 and *C*_2_ = 0.05.

To quantitatively analyze the influence of Wiener constants, the initial optimization wiener constant in HiFi-SIM (denoted *w*_1_) was set to 0.9 and 1.2; the deconvolution wiener constants of fairSIM (denoted Wiener parameter) and HiFi-SIM (denoted *w*_2_) were set to 0.005, 0.01, 0.05, 0.1, 0.2, 0.3, 0.4, and 0.5, respectively. SoftWoRx only set a wiener constant of 0.005 for reconstruction. Ten different regions of interest (ROIs) (53 × 53 pixels), containing ring structures, from the SR images (Fig. S10b) were selected to calculate the SSIM values between ROI images and corresponding ground-truth images (Fig. 3f).

## Supplementary information

Supplementary Information

Supplementary code, test data, and User guide
