# Supplementary material for: High-fidelity structured illumination microscopy by point-spread-function engineering
Source: Light Sci Appl. 2021 Apr 1;10:70. doi: 10.1038/s41377-021-00513-w (PMC8016956; doi:10.1038/s41377-021-00513-w)
Supplement: Supplementary file 1 — Supplementary Information [file 41377_2021_513_MOESM1_ESM.pdf]

Supplementary Information for High-fidelity structured illumination microscopy by point-spread-function engineering

Gang Wen[1,2], Simin Li[1], Linbo Wang[1], Xiaohu Chen[1], Zhenglong Sun[1], Yong Liang[1], Xin Jin[1], Yifan Xing[3], Yaming Jiu[3], Yuguo Tang[*1] & Hui Li[*1]

Correspondence: Y.T (tangyg@sibet.ac.cn) or H.L. (hui.li@sibet.ac.cn)

[1] Jiangsu Key Laboratory of Medical Optics, CAS Center for Excellence in Molecular Cell Science, Suzhou Institute of Biomedical Engineering and Technology, Chinese Academy of Sciences, Suzhou, Jiangsu 215163, China.

[2] Academy for Engineering and Technology, Fudan University, Shanghai 200433, China.

[3] The Center for Microbes, Development and Health, Key Laboratory of Molecular Virology and Immunology, Institute Pasteur of Shanghai, Chinese Academy of Sciences, Shanghai 200031, China.

Supplementary Note 1 Issues of traditional Wiener-SIM reconstruction

1.1 Traditional Wiener-SIM reconstruction algorithm

Super-resolution (SR) structured illumination microscopy (SIM) is a wide-field imaging method that doubles the spatial resolution of fluorescent images[1]. The final SR image is reconstructed by post-processing image algorithms. Here, we first briefly introduce the principle and implementation of the most widely-used Wiener deconvolution-based SIM algorithms[2] (hereafter called "Wiener-SIM"). We start by considering a two-dimensional (2D) raw SIM data

$$D_{\theta,n}(\mathbf{r}) \cong \left\{S(\mathbf{r})\cdot\left[1+m_\theta\cdot\cos\left(2\pi\mathbf{k}_{\theta,ex}\cdot\mathbf{r}+\phi_\theta+\frac{2\pi(n\text{-}1)}{3}\right)\right]\right\}\otimes PSF_{det}(\mathbf{r})+B(\mathbf{r})+N(\mathbf{r}) \quad (1)$$

where subscripts θ and n represent the orientation and phase of the illumination pattern (θ = 1, 2, 3; n = 1, 2, 3); $\mathbf{r}$ is the image spatial coordinates; $S(\mathbf{r})$ represents fluorophore density distribution within specimen, which is illuminated by the structured patterns with sinusoidal intensity; m_θ, $\mathbf{k}_{\theta,ex}$, and ϕ_θ are the modulation depth, pattern wave vector and initial phase of the illumination pattern, respectively; symbol $\otimes$ denotes the convolution operation; $PSF_{det}(\mathbf{r})$ is the actual point spread function (PSF) of the detection objective, $B(\mathbf{r})$ is the background signal, including the out-of-focus fluorescence by excitation illumination and sample scattering, as well as the constant offset of camera, $N(\mathbf{r})$ is noise from both sample and camera. Note that $B(\mathbf{r})$ is not or weakly modulated by structured illumination patterns in different SIM modalities (such as TIRF-SIM, GI-SIM, or conventional 2D-SIM). Here $B(\mathbf{r})$ and $N(\mathbf{r})$ are written explicitly to explain the artifacts issues in SIM.

In the frequency domain, the spectrum of raw SIM data can be obtained by Fourier transform

$$\tilde{D}_{\theta,n}(\mathbf{k}) \cong \left[\frac{m_\theta}{2}\tilde{S}(\mathbf{k}-\mathbf{k}_{\theta,ex})e^{j\left(\phi_\theta+\frac{2\pi(n\text{-}1)}{3}\right)}+\tilde{S}(\mathbf{k})+\frac{m_\theta}{2}\tilde{S}(\mathbf{k}+\mathbf{k}_{\theta,ex})e^{-j\left(\phi_\theta+\frac{2\pi(n\text{-}1)}{3}\right)}\right]\tilde{H}_{det}(\mathbf{k})+\tilde{B}(\mathbf{k})+\tilde{N}(\mathbf{k}) \quad (2)$$

where $\tilde{H}_{det}(\mathbf{k})$ is the actual optical transfer function (OTF) of the microscope, which is the Fourier transform of $PSF_{det}(\mathbf{r})$. $\tilde{S}(\mathbf{k})$ is the spectrum of the sample signal, and $\tilde{S}(\boldsymbol{k}\pm\boldsymbol{k}_{\theta,ex})$ represent the spectrum components that contain the unresolvable high-frequency signals. $\tilde{B}(\mathbf{k})$ and $\tilde{N}(\mathbf{k})$ are the spectrum of the background signal and noise signal, respectively.

For 2D-SIM, the raw SIM data of three different phases are usually collected in one orientation, and all the spectrum components in Eq. (2) can be separated by solving the following equation

$$\begin{bmatrix}\tilde{S}_{\theta,0}(\mathbf{k})\\ \tilde{S}_{\theta,+1}(\mathbf{k}-\mathbf{k}_{\theta,ex})\\ \tilde{S}_{\theta,\text{-}1}(\mathbf{k}+\mathbf{k}_{\theta,ex})\end{bmatrix}=\begin{bmatrix}1 & \frac{m_\theta}{2}e^{j\phi_\theta} & \frac{m_\theta}{2}e^{-j\phi_\theta}\\ 1 & \frac{m_\theta}{2}e^{j\left(\phi_\theta+\frac{2\pi}{3}\right)} & \frac{m_\theta}{2}e^{-j\left(\phi_\theta+\frac{2\pi}{3}\right)}\\ 1 & \frac{m_\theta}{2}e^{j\left(\phi_\theta+\frac{4\pi}{3}\right)} & \frac{m_\theta}{2}e^{-j\left(\phi_\theta+\frac{4\pi}{3}\right)}\end{bmatrix}^{-1}\cdot\begin{bmatrix}\tilde{D}_{\theta,1}(\mathbf{k})\\ \tilde{D}_{\theta,2}(\mathbf{k})\\ \tilde{D}_{\theta,3}(\mathbf{k})\end{bmatrix} \quad (3)$$

where $\tilde{S}_{\theta,0}(\mathbf{k})$ is the 0-order separated spectrum component, and $\tilde{S}_{\theta,+1}(\mathbf{k}-\mathbf{k}_{\theta,ex})$ and $\tilde{S}_{\theta,\text{-}1}(\mathbf{k}+\mathbf{k}_{\theta,ex})$ are the ±1-order separated spectrum components.

To separate and shift the spectrum components accurately, the illumination pattern parameters of $\mathbf{k}_\theta$, m_θ, and ϕ_θ need to be precisely determined from the raw SIM data. Generally, these pattern parameters can be estimated using cross-correlation-based methods[2-6]. In this paper, we implemented an amplitude normalized cross-correlation method combined with the spectrum notch to enable automatic estimation of the illumination pattern parameters (**Supplementary Note 2**).

Upon the separation of different frequency components, the spectrum of the background fluorescent and noise signals are allocated into different frequency components accordingly, given by

$$\begin{cases} \tilde{S}_{\theta,0}(\mathbf{k}) = \tilde{S}(\mathbf{k}) \cdot \widetilde{H}_{det}(\mathbf{k}) + \left[\tilde{B}_0(\mathbf{k}) + \tilde{N}_0(\mathbf{k})\right] \\ \tilde{S}_{\theta,+1}\left(\mathbf{k} - \mathbf{k}_{\theta,ex}\right) = \tilde{S}\left(\mathbf{k} - \mathbf{k}_{\theta,ex}\right) \cdot \widetilde{H}_{det}(\mathbf{k}) + \left[\tilde{B}_{+1}(\mathbf{k}) + \tilde{N}_{+1}(\mathbf{k})\right] \\ \tilde{S}_{\theta,-1}\left(\mathbf{k} + \mathbf{k}_{\theta,ex}\right) = \tilde{S}\left(\mathbf{k} + \mathbf{k}_{\theta,ex}\right) \cdot \widetilde{H}_{det}(\mathbf{k}) + \left[\tilde{B}_{-1}(\mathbf{k}) + \tilde{N}_{-1}(\mathbf{k})\right] \end{cases} \tag{4}$$

where $\tilde{B}_l(\mathbf{k})$ and $\tilde{N}_l(\mathbf{k})$ represent the residual background signal and noise signal contained in the separated spectrum components, respectively. Here, l represents the separated spectrum component orders (l = -1, 0, +1). Note that the residual background in $\tilde{S}_{\theta,0}(\mathbf{k})$ is mainly concentrated in the center of this separated term; for the residual background in $\tilde{S}_{\theta,+1}\left(\mathbf{k} - \mathbf{k}_{\theta,ex}\right)$ and $\tilde{S}_{\theta,-1}\left(\mathbf{k} + \mathbf{k}_{\theta,ex}\right)$, the part not modulated by structured illumination pattern is mainly concentrated in the center of the separated terms, while the part modulated by structured illumination pattern is shifted along the pattern wave vector $\mathbf{k}_{\theta,ex}$.

Then, as in [2, 3, 5], these separated components can be shifted back to their correct position. Note that the residual background signal and noise signal in Eq. (4) are also moved synchronously, so the shifted components can be denoted as

$$\tilde{C}_0(\mathbf{k}) = \tilde{S}(\mathbf{k}) \cdot \widetilde{H}_{det}(\mathbf{k}) + \left[\tilde{B}_0(\mathbf{k}) + \tilde{N}_0(\mathbf{k})\right] \tag{5}$$

$$C_{+1}\left(\mathbf{k} + \mathbf{k}_{\theta,ex}\right) \cong \tilde{S}(\mathbf{k}) \cdot \widetilde{H}_{det}\left(\mathbf{k}+\mathbf{k}_{\theta,ex}\right) + \left[\tilde{B}_{+1}\left(\mathbf{k}+\mathbf{k}_{\theta,ex}\right) + \tilde{N}_{+1}\left(\mathbf{k}+\mathbf{k}_{\theta,ex}\right)\right] \tag{6}$$

$$\tilde{C}_{-1}\left(\mathbf{k} - \mathbf{k}_{\theta,ex}\right) \cong \tilde{S}(\mathbf{k}) \cdot \widetilde{H}_{det}\left(\mathbf{k} - \mathbf{k}_{\theta,ex}\right) + \left[\tilde{B}_{-1}\left(\mathbf{k} - \mathbf{k}_{\theta,ex}\right) + \tilde{N}_{-1}\left(\mathbf{k} - \mathbf{k}_{\theta,ex}\right)\right] \tag{7}$$

In Eq. (5), the residual background spectrum remains in the center of $\tilde{C}_0(\mathbf{k})$; In Eqs. (6,7), the residual background modulated by the structured illumination pattern is shifted to the same position as the residual background in $\tilde{C}_0(\mathbf{k})$, while the part not modulated by the structured illumination pattern is shifted to the center of $C_{+1}\left(\mathbf{k} + \mathbf{k}_{\theta,ex}\right)$ and $\tilde{C}_{-1}\left(\mathbf{k} - \mathbf{k}_{\theta,ex}\right)$. As a result, the residual background signal at the center of the reconstructed spectrum causes the reconstructed SIM image contain obvious **background fluorescence**, while the residual background spectrum corresponding to the centers of $C_{+1}\left(\mathbf{k} + \mathbf{k}_{\theta,ex}\right)$ and $\tilde{C}_{-1}\left(\mathbf{k} - \mathbf{k}_{\theta,ex}\right)$ results in **periodic honeycomb artifacts**[7]. In addition, the residual high-frequency noise signals in Eqs. (6,7) can cause **random discontinuous artifacts**, such as **hammerstroke artifacts**, in the reconstructed images.

Conventionally, to suppress the residual background signals in the center of all shifted spectrum components, the OTF attenuation method[3,5,8] is generally applied on each shifted component. In the traditional Wiener-SIM we implemented, a Gaussian function is usually employed to implement OTF attenuation

$$\tilde{g}\left(\mathbf{k} + L \cdot \mathbf{k}_{\theta,ex}\right) = 1\text{-}attStrength \cdot e^{-\frac{\left(\mathbf{k}+L\cdot\mathbf{k}_{\theta,ex}\right)^2}{(0.5\cdot attWidth)^2}} \tag{8}$$

where $attStrength$ and $attWidth$ are the attenuation strength and width, respectively, which are constant values empirically determined based on the quality of raw SIM data. L represents the shifted component orders (In this paper, for 2D-SIM data: L = -1, 0, +1; for three-dimensional (3D) SIM data: L = -2, -1, 0, +1, +2).

Thus, a directly-combined spectrum by weighted sum of all the shifted components can be obtained

$$\tilde{S}_{directly\text{-}combined}(\mathbf{k}) = \sum_\theta \tilde{g}\left(\mathbf{k} + L \cdot \mathbf{k}_{\theta,ex}\right) \cdot \tilde{C}_L\left(\mathbf{k} + L \cdot \mathbf{k}_{\theta,ex}\right) \cdot \widetilde{H}^*_{use}\left(\mathbf{k} + L \cdot \mathbf{k}_{\theta,ex}\right) \tag{9}$$

where $\widetilde{H}_{user}$ represents the OTF provided by the user for the reconstruction algorithm. Symbol * denotes the conjugate operation. Substituting Eqs. (5–7) into Eq. (9), the directly-combined spectrum can be expressed as

$$\tilde{S}_{directly\text{-}combined}(\mathbf{k}) \cong \sum_\theta \{\tilde{g}(\mathbf{k}+L\cdot\mathbf{k}_{\theta,ex})\cdot\tilde{S}(\mathbf{k})\cdot[\widetilde{H}_{det}(\mathbf{k}+L\cdot\mathbf{k}_{\theta,ex})\cdot\widetilde{H}^*_{use}(\mathbf{k}+L\cdot\mathbf{k}_{\theta,ex})] + \tilde{g}(\mathbf{k}+L\cdot\mathbf{k}_{\theta,ex})\cdot[\tilde{B}'(\mathbf{k}+L\cdot\mathbf{k}_{\theta,ex})+\tilde{N}'(\mathbf{k}+L\cdot\mathbf{k}_{\theta,ex})]\} \quad (10)$$

In Eq. (10), the weight $\tilde{g}$ not only attenuates the residual background signal spectrum, but it also simultaneously attenuates the real sample signal spectrum. In addition, it is worth noting that $\widetilde{H}_{det}(\mathbf{k})$ **is the actual OTF that matches the imaging conditions during the raw data acquisition, while** $\widetilde{H}_{use}(\mathbf{k})$ **is usually generated by experimental measurements or mathematical models.**

Thereafter, deconvolution and apodization are applied to increase the spatial resolution and suppress high-frequency related artifacts. For traditional Wiener-SIM, the reconstructed spectrum can be obtained by the generalized Wiener filter deconvolution[2]

$$\tilde{S}_{SIM}(\mathbf{k}) = \tilde{S}_{directly\text{-}combined}(\mathbf{k})\cdot\widetilde{W}(\mathbf{k}) \quad (11)$$

$$\widetilde{W}(\mathbf{k}) = \frac{\tilde{A}(\mathbf{k})}{\sum_\theta \tilde{g}(\mathbf{k}+L\cdot\mathbf{k}_{\theta,ex})\cdot|\widetilde{H}_{use}(\mathbf{k}+L\cdot\mathbf{k}_{\theta,ex})|^2+w^2} \quad (12)$$

where $\tilde{A}(\mathbf{k})$ is the apodization function. w is the deconvolution Wiener constant, which is an empirical value. In the traditional Wiener-SIM we implemented, $\widetilde{H}_{use}(\mathbf{k})$ is an approximate OTF generated based on the parameters of the detection objective and emission wavelength[5,9]

$$\widetilde{H}_{use}(\mathbf{k}) = \frac{2b(|\mathbf{k}|)\text{-}\sin[2b(|\mathbf{k}|)]}{\pi} \quad (13)$$

where $b(\mathbf{k}) = \cos^{-1}\left(\frac{\mathbf{k}}{\mathbf{k}_c}\right)$, $\mathbf{k}_c = \frac{2NA}{\lambda_{em}}$ is the cutoff frequency of the imaging system; NA is the numerical aperture (NA) of the detection objective; and λ_{em} is the emission wavelength. Apodization function $\tilde{A}(\mathbf{k})$ has the same form as $\widetilde{H}_{user}(\mathbf{k})$, but the cutoff frequency is extended to $\mathbf{k}_{\theta,ex}+\mathbf{k}_c$. To properly compensate for the deviation of $\widetilde{H}_{use}(\mathbf{k})$ relative to $\widetilde{H}_{det}(\mathbf{k})$, a modified model of OTF is generally used[5,10]

$$\widetilde{H}'_{use}(\mathbf{k}) = \widetilde{H}_{use}(\mathbf{k})\cdot\beta^{|\mathbf{k}|} \quad (14)$$

where β is a "dampening" factor for compensating aberrations, which is an empirical value. In the traditional Wiener-SIM implemented by us, β ranges from 0 to 1.

Finally, the reconstructed SR image can be obtained as

$$I_{SIM}(\mathbf{r}) = F^{-1}[\tilde{S}_{SIM}(\mathbf{k})] \quad (15)$$

where $F^{-1}\{\cdot\}$ denotes the inverse Fourier transform.

1.2 Knotty issues in traditional Wiener-SIM reconstruction

As discussed above, traditional Wiener-SIM estimates the illumination pattern parameters from the raw data, and then reconstructs SR images based on the generalized Wiener filtering deconvolution procedure (Eqs. (9–15)). Many efforts have been made to pursue high-quality SR images with minimal artifacts, including in-depth imaging system establishment protocol[11], accurate reconstruction parameter estimation[2-6], practical guidelines for data acquisition and sample preparation[7,12], fine tuning of user-defined parameters[10], and some open-source reconstruction tools[5,6,13,14]. Even so, reconstruction

artifacts still cannot be avoided in practice[15].

1.2.1 Typical artifacts in SR-SIM images

Reconstruction artifacts in SR-SIM images have been discussed extensively recently. A few summaries can be found in published papers[6,7,10]. Although some kinds of artifacts can be distinguished subjectively, it is difficult to robustly eliminate them. Artifacts frequently appear in the SR-SIM images even in publications[5,7,15].

Artifacts related to parameter estimation and high-frequency noise. Generally, raw data with high modulation depths and high signal-to-noise ratio (SNR) is beneficial for estimating correct reconstruction parameters and reducing reconstruction artifacts[2,6,7,16-18]. For suboptimal raw data with low SNR, strong background fluorescence, or low modulation contrast, traditional Wiener-SIM may not be able to estimate the correct pattern vectors, resulting in **hatching artifacts**[7]. Even if the correct pattern vectors can be determined, the pattern modulation depths estimated from these suboptimal data are usually smaller than the actual values. Smaller modulation factors will cause the high-order shifted components in the recombined spectrum to be over-amplified, causing **snowflake-like** or **sidelobe artifacts**[6,7,10]. Moreover, as the high-frequency noise in the recombined spectrum can be amplified synchronously (Eqs. (6, 7, 12)), the random discontinuous artifacts, such as **hammerstroke artifacts,** in the reconstructed image become serious[7].

Artifacts associated with the abnormal spectrum. Even if the correct reconstruction parameters are estimated, SR-SIM images reconstructed by traditional Wiener-SIM may still suffer from artifacts. As shown in Eqs. (6,7), the residual background signal spectrum at the center of the high-order shifted components often causes **honeycomb artifacts**. Moreover, a too-small wiener constant can cause **hammerstroke artifacts** because the high-frequency noise spectrum cannot be effectively suppressed[10]. In addition, another cause of **sidelobe artifacts** is the patchy features of the reconstructed spectrum that may be caused by OTF mismatch[10].

In general, the reconstruction of suboptimal data is more prone to artifacts. However, in practice, a large amount of suboptimal data is often inevitably collected in SIM imaging. To avoid the risk of artifacts, these suboptimal raw data are not effectively utilized or even abandoned. This not only causes a waste of time and money in imaging experiments, but more importantly limits the wide application of SR-SIM in some special situations, such as samples with strong background fluorescence or suboptimal modulation contrast.

1.2.2 Trade-off between eliminating artifacts and protecting fine sample structures

As discussed above, OTF attenuation method is generally employed in traditional Wiener-SIM to suppress artifacts and improve optical sectioning (OS) (Eq. (8)). However, as shown in Eq. (10), OTF attenuation can simultaneously attenuate the residual out-of-focus signal and the real sample signal. Thus, **there is a trade-off between eliminating artifacts and protecting fine and weak structures** (Fig. 5). If the attenuation strength is small, the reconstructed images may still contain obvious residual background fluorescence and related artifacts. Conversely, as the attenuation strength increases, some fine and weak structures in the reconstructed images may be attenuated or even disappear completely[5].

Properly increasing the wiener constant can also reduce certain reconstruction artifacts[7]. However, as the wiener constant increases, the fine structures in the reconstructed image become blurred due to the attenuation of high-frequency signals, and some weak sample features may also be attenuated or even lost in the reconstructed images[5]. Therefore, it is generally recommended to set a smaller wiener constant to ensure the spatial resolution and avoid losing the real sample features as much as possible,

at the cost of possible artifacts.

1.2.3 Trade-off between super-resolution and optical sectioning in 2D-SIM

For 2D-SIM imaging, out-of-focus background is the main challenge that limits the SR image quality in addition to reconstruction artifacts[18]. In terms of imaging principle, due to the missing-cone problem of optical transfer function (OTF)[2], **there is a trade-off between optimal OS performance and doubling the lateral resolution.** Thus, 2D-SIM images with the best lateral resolution usually show weak OS performance[18-24]. 3D-SIM overcomes this disadvantage and obtains twice the lateral and axial resolution simultaneously, but the slow imaging speed makes it unsuitable for fast and long-term dynamic imaging. In terms of algorithm implementation, using 2D OTF to reconstruct 2D data of 3D samples (both two-beam interference data and single-slice three-beam interference data), the "Wiener deconvolution" solution in Eq. **(**12**)** is one of the important factors limiting the sectioning capability of 2D Wiener-SIM algorithms. As far as we know, there is a strong convex peak in the center of $\widetilde{W}(\mathbf{k})$ in Eq. (12), which can enhance the residual out-of-focus spectrum at the center of reconstructed spectrum $\tilde{S}_{SIM}(\mathbf{k})$ in Eq. (11). Therefore, SR images reconstructed by 2D Wiener-SIM usually contain obvious residual background fluorescence and related artifacts (Figs. S8b and S15–S17). In addition, for single-slice 3D-SIM data, SR images reconstructed by 2D Wiener-SIM usually contain more residual out-of-focus background than those reconstructed by full 3D-SIM reconstruction (Fig. S18). To date, many methods have been developed to improve the OS performance of 2D-SIM[5,20-24]. However, most of these methods, such as OTF attenuation and MAP-SIM[20], cannot well balance the trade-off between suppressing out-of-focus background and preserving fine and weak structures (Figs. 5 and S14).

1.2.4 Effect of PSF mismatch in Wiener-SIM

PSF represents the actual response of the imaging system to the fluorescent point source. As shown in Eqs. (9–12), the reconstruction process of Wiener-SIM involves the actual OTF of the imaging system ($\widetilde{H}_{det}(\mathbf{k})$) and the OTF provided by the user ($\widetilde{H}_{use}(\mathbf{k})$). Therefore, using a PSF that matches the actual imaging conditions during the raw data acquisition is another crucial factor to ensure high-quality SR images by Wiener-SIM[2,7,10,17]. Typically, the equivalent OTF of SR-SIM obtained by directly combining the shifted spectrum components will generate patchy features, resulting in that the corresponding PSF also contains strong sidelobes (Fig. 1a-c). As a result, the reconstructed SR-SIM image before deconvolution has strong sidelobe artifacts parallel to the real image (Fig. S1a,d,g). If the PSF used for deconvolution matches the actual imaging conditions, the "Wiener deconvolution" procedure could correct the abnormal features, and obtain SR images with minimal artifacts. (Fig. S7). However, once the adopted PSF does not match, the reconstructed spectrum obtained by "Wiener deconvolution" procedure will remain abnormal features, which result in artifacts in the final SR image (Figs. 2 and S4a,b). Therefore, in SIM imaging, it is usually emphasized to measure the actual PSF for the reconstruction algorithms to avoid artifacts. However, calibrating the actual PSF is a complex and professional task[7], which is hardly done by regular users.

1.2.5 Complex adjustment of multiple user-defined parameters

In addition to the experimental inputs, including the raw SIM data and the adopted PSF/OTF, the quality of SR-SIM images depends on multiple user-defined reconstruction parameters[10,25]. For Wiener-SIM, there are many user-defined parameters, including Wiener filter parameter, apodization parameters, zero order suppression parameters, OTF correction parameter, which need to be manually adjusted during the reconstruction. These typical parameters were well summarized in the recently

published paper[10]. Unreasonable user-defined parameters can also cause reconstruction artifacts. Generally, users need to adjust the user-defined parameters according to the characteristics and quality of the raw data. For ordinary users, the effects of these user-defined parameters and the quality of the reconstructed images remain a “black box”. Although some protocols for guiding the setting and adjustment of user-defined parameters exist[10,26], the professional and complicated optimization process limits the accessibility of ordinary users and biologists.

In summary, the reconstruction of SR-SIM still faces the challenges of the above-mentioned knotty issues due to the lack of simple, effective, and general solutions. Currently, Wiener-SIM-based algorithms have high requirements on the raw SIM data, the PSF used, and user-defined parameters, which complicate the reconstruction of SR-SIM, and may destroy the confidence of most ordinary users and biologists in SR-SIM technology. Therefore, it is critical to develop a robust and easy-to-operate high-fidelity SIM algorithm to solve these knotty issues in SIM reconstruction.

Supplementary Note 2 High-fidelity SIM reconstruction algorithm based on PSF engineering

To overcome the above-mentioned problems in traditional Wiener-SIM reconstruction, we have developed a high-fidelity SIM reconstruction algorithm (hereinafter referred to as “HiFi-SIM”) for reconstructing high-quality SR-SIM images with minimal artifacts. HiFi-SIM solves these knotty issues by optimizing the illumination parameter estimation algorithm and the reconstructed spectrum recombination algorithm. The details are discussed below.

2.1 Automatic determination of illumination pattern parameters

Accurate estimation of the illumination pattern parameters from raw SIM data is essential to reconstruct high-quality SR-SIM images. Small deviations of reconstruction parameters may cause noticeable reconstructed artifacts[7,10,17]. To our knowledge, the “cross-correlation-based” methods were used most widely for parameter estimation[2-6,13,14]. The methods calculate the cross-correlation map of the 0-order separated spectrum and the 1-order separated spectrum to determine the pattern wave vectors. However, as these spectrum components are always attenuated by the OTF of the imaging system, the peaks associated with the pattern wave vector may be very weak in the cross-correlation map[27], often lower than the values in the low-frequency region (Fig. S2a). Thus, a MASK is generally used to define the fitting region of the peak position of the pattern vectors[5,13]. The radius of the MASK usually needs to be manually determined or even repeatedly adjusted to search for the correct peak position, especially for TIRF-SIM data where the spatial frequency of the excitation pattern is close to the cutoff frequency of the imaging system (Fig. S2a,b). For samples with obvious periodic structures, in particular, the local periodicities of the sample structures and the illumination pattern wave vectors are difficult to be distinguished with the current cross-correlation methods[3,4] (Fig. S2d,e).

To address the above problems, we developed a modified method to determine the pattern vectors by the combination of amplitude normalized cross-correlation[6,28] with a spectrum notch (Fig. S1a), including two steps of raw data preprocessing and cross-correlation calculation.

Step 1: Raw data preprocessing. To enhance the peaks of pattern wave vectors in the cross-correlation map and remove certain out-of-focus background and noise, Richardson–Lucy (RL) deconvolution is first performed on raw SIM data[27,29], and default number of iterations is set as 5. Specifically, the PSF used for deconvolution is an approximate PSF obtained by the inverse Fourier transform of Eq. (13) to avoid complicated system calibration.

Step 2: Automatic reconstruction parameter estimation. To reduce the contribution of the low-frequency signals of the initially separated spectrum components and the local-periodicity of the sample structures to the cross-correlation map, a notch filter was designed to attenuate the separated 0-order and 1-order spectrum components as

$$\tilde{F}_{Notch}(\mathbf{k}) = \begin{cases} 1\text{-}e^{-\frac{\mathbf{k}^4}{2\cdot(0.5\mathbf{k}_c)^4}}, & |\mathbf{k}| \leq \mathbf{k}_c \\ 0, & |\mathbf{k}| > \mathbf{k}_c \end{cases} \quad (16)$$

where $\mathbf{k}_c$ is the cutoff frequency of the imaging system in reciprocal space. Then, the processed spectrum components are normalized by their own maximum amplitude. Finally, the cross-correlation map is again multiplied by the notch filter to further suppress the contribution of the low-frequency

region. Besides the above difference, the cross-correlation calculation follows the procedure in fairSIM[5].

In the filtered cross-correlation map, the peaks associated with the pattern vectors become most prominent which can be automatically determined. In addition, our method can automatically determine the correct pattern vectors from the majority of biological sample data, including low SNR data (Figs. S21-S23), TIRF-SIM data (Fig. S2c), and even data with obvious periodic structures (Fig. S2f).

2.2 Spectrum optimization of HiFi-SIM

As discussed in **Supplementary Note 1**, there are still many knotty issues in traditional Wiener-SIM reconstruction. For these reasons, even if the reconstruction parameters are correctly determined, SR images reconstructed by Wiener-SIM-based algorithms may still suffer from various artifacts[6,7,10,25]. We note that the root cause of this barrier is the abnormal features of the equivalent OTF of SR-SIM[15,18,30] ($\widetilde{H}_{Theoretical}(\mathbf{k})$ in Fig. 1a). Essentially, OTF determines the relative strength of different frequencies in the reconstructed spectrum, so the abnormal features of $\widetilde{H}_{Theoretical}(\mathbf{k})$ are usually characterized simultaneously as the abnormalities in the directly-combined spectrum $\tilde{S}_{directly\text{-}combined}(\mathbf{k})$ (Eq. (9); Figs. 1b,c and S1a), and these abnormal spectrums are generated as artifacts in reconstructed images (Figs. 1b,c and S1d,g). For example, honeycomb artifacts correspond to multiple pairs of sharp residual background signal spectrum peaks in the center of the ±1-order shifted components (region A in Fig. S3a). Sidelobe artifacts correspond to the patchy features in the reconstructed spectrum (region B in Fig. S3a). The residual out-of-focus background of the reconstructed image correspond to the raised peak in the center of the reconstructed spectrum (region C in Fig. S3a). In addition, random discontinuous artifacts, such as hammerstroke artifacts, correspond to high-frequency noise in the reconstructed spectrum. This means that correcting the abnormal features of the equivalent OTF can eliminate the abnormalities in $\tilde{S}_{directly\text{-}combined}(\mathbf{k})$, thereby suppressing reconstruction artifacts. To solve these knotty issues in Wiener-SIM-based algorithms, HiFi-SIM performs a two-step spectrum optimization method based on PSF engineering to optimize $\tilde{S}_{directly\text{-}combined}(\mathbf{k})$.

First, we develop an initial optimization function $\widetilde{W}_1(\mathbf{k})$ to correct the abnormalities of $\tilde{S}_{directly\text{-}combined}(\mathbf{k})$. It is worth pointing out that the ideal form of OTF for SR-SIM ($\widetilde{H}_{ideal}(\mathbf{k})$ in Fig. 1a) should be same form of that for wide-field imaging ($\widetilde{H}_{det}(\mathbf{k})$ in Fig. 1a) with the cut-off frequency extended to $(\boldsymbol{k}_c + \boldsymbol{k}_{ex})$, where $\boldsymbol{k}_c$ is the cut-off frequency of wide-field imaging and $\boldsymbol{k}_{ex}$ is spatial frequency of the excitation pattern, so that the imaging result could be a perfect image in principle. Inspired by this, the optimization of $\tilde{S}_{directly\text{-}combined}(\mathbf{k})$ is equivalent to correcting the equivalent OTF $\widetilde{H}_{Theoretical}(\mathbf{k})$ to the ideal form $\widetilde{H}_{ideal}(\mathbf{k})$ using $\widetilde{W}_1(\mathbf{k})$, as follows

$$\widetilde{\mathrm{H}}_{\mathrm{ideal}}(\boldsymbol{k}) \cong \widetilde{H}_{Theoretical}(\mathbf{k}) \cdot \widetilde{W}_1(\mathbf{k}) \tag{17}$$

where

$$\widetilde{H}_{ideal}(\mathbf{k}) = \widetilde{H}_{det}\left(\frac{\mathbf{k}_c}{\mathbf{k}_c+\mathbf{k}_{ex}} \cdot \mathbf{k}\right) \tag{18}$$

$$\widetilde{H}_{Theoretical}(\mathbf{k}) = \frac{m_{ex}}{2}\widetilde{H}_{det}(\mathbf{k}+\mathbf{k}_{ex}) + \widetilde{H}_{det}(\mathbf{k}) + \frac{m_{ex}}{2}\widetilde{H}_{det}(\mathbf{k}-\mathbf{k}_{ex}) \tag{19}$$

where m_{ex} is the modulation depth of the excitation patterns. Theoretically, as shown in Eqs. (18,19),

it is optimal to use the actual OTF of the imaging system ($\widetilde{H}_{det}(\mathbf{k})$) to generate $\widetilde{H}_{ideal}(\mathbf{k})$ and $\widetilde{H}_{Theoretical}(\mathbf{k})$, because this helps to avoid OTF mismatch problems.

In HiFi-SIM, to avoid complex and professional OTF calibration work, a theoretical OTF in Eq. (14) ($\widetilde{H}_{use}(\mathbf{k})$) is employed instead of the actual OTF. Therefore, according to the frequency domain Wiener filtering[31], $\widetilde{\mathrm{W}}_1(\boldsymbol{k})$ is designed as the following mathematical formula

$$\widetilde{\mathrm{W}}_1(\boldsymbol{k}) = \frac{\widetilde{H}_{use}\left(\frac{\mathbf{k}_C}{\mathbf{k}_C+\mathbf{k}_{\theta,ex}}\cdot\mathbf{k}\right)}{\sum_\theta \tilde{g}_1(\mathbf{k}+L\cdot\mathbf{k}_{\theta,ex})\cdot\left|\widetilde{H}_{use}(\mathbf{k}+L\cdot\mathbf{k}_{\theta,ex})\right|^2+(w_1)^2} \tag{20}$$

$$\tilde{g}_1(\mathbf{k}+L\cdot\mathbf{k}_{\theta,ex}) = \begin{cases} 1\text{-}attStrength\cdot e^{-\frac{(\mathbf{k}+L\cdot\mathbf{k}_{\theta,ex})^2}{(0.5\cdot attWidth)^2}}, & L = \pm 1 \\ 1, & L = 0 \end{cases} \tag{21}$$

where w_1 is the initial optimization wiener constant, which is an empirical value; and $attStrength$ and $attWidth$ are the attenuation strength and width, respectively, which are empirical constant values determined according to the quality of raw data. Here, the power spectrum $\left|\widetilde{H}_{use}(\mathbf{k}+L\cdot\mathbf{k}_{\theta,ex})\right|^2$ instead of $\left|\widetilde{H}_{use}(\mathbf{k}+L\cdot\mathbf{k}_{\theta,ex})\right|$ is used in the denominator to avoid errors arising from coordinates of $\pm\mathbf{k}_{\theta,ex}$ not satisfying the "Fourier shift theorem"[2,14]. Similar to the traditional Wiener deconvolution solution (Eqs. (8, 12)), the weight function $\tilde{g}_1(\mathbf{k}+L\cdot\mathbf{k}_{\theta,ex})$ is also used to modulate the raised peaks at the center of the shifted OTFs. The difference is that the weight of the 0-order component in $\tilde{g}_1(\mathbf{k}+L\cdot\mathbf{k}_{\theta,ex})$ is set to 1 to modulate the center of the optimized OTF as an inverted peak (Fig. 1d,e). This overcomes the challenge that $\widetilde{\mathrm{W}}(k)$ in Eq. (12) always enhances the residual out-of-focus background of the reconstructed image, thereby improving the optical sectioning of the reconstruction algorithm. With the initial optimization by $\widetilde{\mathrm{W}}_1(\boldsymbol{k})$, the abnormalities of $\tilde{S}_{directly\text{-}combined}(\mathbf{k})$ can be well corrected (Figs. S3a,b), so the typical artifacts such as sidelobes, honeycombs, and hammerstroke artifacts, as well as the residual background, are effective eliminated in reconstructed images (Fig. S3e,h).

Then, we develop another optimization function $\widetilde{W}_2(\mathbf{k})$ for deconvolution. In the initial optimization step, $\widetilde{H}_{use}(\mathbf{k})$ in $\widetilde{W}_1(\mathbf{k})$ is a theoretical OTF generated based on imaging parameters. In this vein, the mismatch between $\widetilde{H}_{use}(\mathbf{k})$ and the actual OTF ($\widetilde{H}_{det}(\mathbf{k})$) may cause partial abnormal features remaining in the initial optimized spectrum, which is confirmed in Fig. S3b. To overcome the challenge caused by OTF mismatch, $\widetilde{W}_2(\mathbf{k})$ is also generated using $\widetilde{H}_{Theoretical}(\mathbf{k})$ based on Wiener filtering. Specifically, $\widetilde{W}_2(\mathbf{k})$ has the same form as Eq. (12), but the apodization function and weight function used are as follows

$$\tilde{A}(\mathbf{k}) = e^{-\frac{1}{2}\left(\frac{\mathbf{k}}{ApoFWHM}\sqrt{2\ln 2}\right)^2} \tag{22}$$

$$\tilde{g}_2(\mathbf{k}+L\cdot\mathbf{k}_{\theta,ex}) = \begin{cases} 1-\frac{attStrength}{1.15}\cdot e^{-\frac{(\mathbf{k}+L\cdot\mathbf{k}_{\theta,ex})^2}{(0.5\cdot attWidth)^2}}, & L = \pm 1 \\ 1-\frac{attStrength}{1.05}\cdot e^{-\frac{(\mathbf{k}+L\cdot\mathbf{k}_{\theta,ex})^2}{(0.5\cdot attWidth)^2}}, & L = 0 \end{cases} \tag{23}$$

where $ApoFWHM$ is the full width at half maximum (FWHM) of $\tilde{A}(\mathbf{k})$[13]. In HiFi-SIM, the apodization function $\tilde{A}(\mathbf{k})$ is used to suppress artifacts related to high-frequency noise (Fig. S21). The deconvolution wiener constant in $\widetilde{W}_2(\mathbf{k})$ is denoted as w_2, which is also an empirical value. In

addition, similar to the problem in traditional Wiener-SIM (**Supplementary Note 1**), applying OTF attenuation in $\widetilde{W}_1(\mathbf{k})$ may cause fine and weak signals to be attenuated or even lost (Fig. S3e,h). To this end, the weight function $\tilde{g}_2(\mathbf{k}+L\cdot\mathbf{k}_{\theta,ex})$ is used for active compensation the attenuated signals. With the deconvolution optimization by $\widetilde{W}_2(\boldsymbol{k})$, the reconstructed spectrum has almost no obvious abnormal features and the high-frequency signal is significantly enhanced (Fig. S3c), thereby obtaining the high-fidelity SR-SIM image with minimal artifacts (Fig. S3f). In particular, the joint optimization of $\widetilde{W}_1(\boldsymbol{k})$ and $\widetilde{W}_2(\boldsymbol{k})$ can better balance the trade-off between rejecting the out-of-focus blur and protecting fine and weak structures during SIM reconstruction, thus effectively recovering the attenuated or lost real signals in the initial optimization step (Fig. S3h,i).

Finally, the high-fidelity SR-SIM image obtained by spectrum optimization is

$$I_{SIM}(\mathbf{r})=F^{-1}\left[\tilde{S}_{directly\text{-}combined}(\mathbf{k})\cdot\widetilde{W}_1(\mathbf{k})\cdot\widetilde{W}_2(\mathbf{k})\right](\mathbf{r}) \tag{24}$$

The default empirical parameters in HiFi-SIM work well for most tested data with acceptable quality (for two-beam SIM: 3 angles, 3 phases; for three-beam SIM (see Section 2.3 of **Supplementary Note 2**): 3 angles, 5 phases): $\beta=1.0$; $attWidth=1.0$; $ApoFWHM = min\left\{0.5,\frac{0.5\mathbf{k}_{ex}}{\mathbf{k}_c}\right\}$; w_1=1.2; w_2=0.1. Only attenuation strength $attStrength$ needed to be manually set to adjust the OS performance (Fig. S20). See **Supplementary Note 3** for a detailed discussion of the reconstruction parameter settings.

Note that HiFi-SIM is more immune to reconstruction artifacts related to OTF mismatch, compared with Wiener-SIM (Figs. S4 and S5). Therefore, HiFi-SIM can use the theoretical OTF generated based on imaging parameters instead of the actual OTF to reconstruct high-quality SR images, thereby avoiding the complicated and professional work of calibrating the actual OTF.

HiFi-SIM is implemented with a graphical user interface (GUI) of MATLAB software (see **Supplementary Code**).

2.3 HiFi-SIM extension for single-layer 3D-SIM data reconstruction

HiFi-SIM has been extended to reconstruct single-layer 3D-SIM data. For 3D-SIM data, a method similar to that in 2D-SIM is used to determine the reconstruction parameters (see **Supplementary Code**). After determining the reconstruction parameters, as in Eqs. (8,9,14), the directly combined spectrum $\tilde{S}_{directly\text{-}combined}(\mathbf{k})$ can be obtained. The difference is that the spectrum order L = -2, -1, 0, +1, +2, due to the addition of ± 2-order shifted spectrum components.

For single-layer 3D-SIM data reconstruction, the initial optimization function $\widetilde{W}_1(\mathbf{k})$ has the same form as Eqs. (20, 21), and the spectrum components L = -2, -1, 0, +1, +2. Also, in the weight function $\tilde{g}_1(\mathbf{k}+L\cdot\mathbf{k}_{\theta,ex})$, the weights of ±1-order and ±2-order components are the same, while the weight of 0-order component is 1.

Similarly, the deconvolution function $\widetilde{W}_2(\boldsymbol{k})$ has the same form as Eq. (12), and the apodization function in $\widetilde{W}_2(\boldsymbol{k})$ is the same as Eq. (22). The weight function of $\widetilde{W}_2(\boldsymbol{k})$ is as follows

$$\tilde{g}_2(\mathbf{k}+L\cdot\mathbf{k}_{\theta,ex})=\begin{cases}\frac{1}{1.2}\left\{1-attStrength\cdot e^{-\frac{\left(\mathbf{k}+L\cdot\mathbf{k}_{\theta,ex}\right)^2}{(0.5\cdot attWidth)^2}}\right\}, & L=\pm1,\pm2\\ 1-\frac{attStrength}{1.05}\cdot e^{-\frac{\left(\mathbf{k}+L\cdot\mathbf{k}_{\theta,ex}\right)^2}{(0.5\cdot attWidth)^2}}, & L=0\end{cases} \tag{25}$$

In addition, all empirical parameters are the same as those in HiFi-SIM for 2D-SIM data reconstruction, and use the same default values (see Section 2.2 of **Supplementary Note 2** and **Supplementary Note 3**).

2.4 Simulation of equivalent OTFs of directly-combined SR-SIM and HiFi-SIM

The OTFs and PSFs of wide-field imaging, directly-combined SR-SIM, and ideal SR-SIM (Fig. 1a), were calculated from the theoretical OTF of TIRF imaging (Eq. (13)) and TIRF-SIM imaging conditions. The imaging system parameters include excitation wavelength of 488 nm, emission wavelength of 525 nm, oil objective with NA of 1.49, and the single pixel size of 65 nm/pixel. The theoretical OTF of directly combined SR-SIM as in Eq. (19)[18,30]. For the simulation of Fig. 1, the modulation depth of the excitation pattern $m_{ex} = 1.0$. In addition, the ideal OTF of SR-SIM is given in Eq. (18).

For 2D-SIM, the equivalent OTF of the directly combined spectrum $\tilde{S}_{directly\text{-}combined}(\mathbf{k})$ is generated based on the power spectrum of theoretical OTF (Fig. 1b,c)

$$\widetilde{H}_{directly\text{-}combined}(\mathbf{k}) = \sum_{\theta} \left\{ \frac{\widetilde{H}_{use}(\mathbf{k}+\mathbf{k}_{\theta,ex})\widetilde{H}_{use}{}^{*}(\mathbf{k}+\mathbf{k}_{\theta,ex})}{2} + \widetilde{H}_{use}(\mathbf{k})\widetilde{H}_{use}{}^{*}(\mathbf{k}) + \frac{\widetilde{H}_{use}(\mathbf{k}-\mathbf{k}_{\theta,ex})\widetilde{H}_{use}{}^{*}(\mathbf{k}-\mathbf{k}_{\theta,ex})}{2} \right\} \tag{26}$$

where θ represents the orientation of illumination pattern ($\theta = 1, 2, 3$).

As shown in Eq. (9), optimizing $\tilde{S}_{directly\text{-}combined}(\mathbf{k})$ is equivalent to optimizing its equivalent OTF $\widetilde{H}_{directly\text{-}combined}(\mathbf{k})$, so the equivalent OTF of HiFi-SIM (Fig. 1d) can be expressed as

$$\widetilde{H}_{HiFi\text{-}SIM}(\mathbf{k}) = \widetilde{H}_{directly\text{-}combined}(\mathbf{k}) \cdot \widetilde{W}_1(\mathbf{k}) \cdot \widetilde{W}_2(\mathbf{k}) \tag{27}$$

With the joint optimization by $\widetilde{W}_1(\mathbf{k})$ and $\widetilde{W}_2(\mathbf{k})$, the equivalent OTF of HiFi-SIM with relative uniform axial and circumferential distribution can be obtained, thereby realizing perfect SR-SIM with little artifacts (Fig. 1d-f). It is worth noting that $\widetilde{H}_{HiFi\text{-}SIM}(\mathbf{k})$ presents a downward central depression, ensuring that HiFi-SIM has better OS capability than Wiener-SIM.

Supplementary Note 3 Guidelines for parameter setting in HiFi-SIM

HiFi-SIM can be used for reconstructing raw SIM data collected on commercial setups and custom-built setups. Here, we suggested an optimization process to guide users to reconstruct high-quality SR-SIM images from raw data with different qualities using HiFi-SIM. HiFi-SIM provides a **Simple mode** and a **Professional mode** to meet the reconstruction of raw data with high-quality and suboptimal quality, respectively.

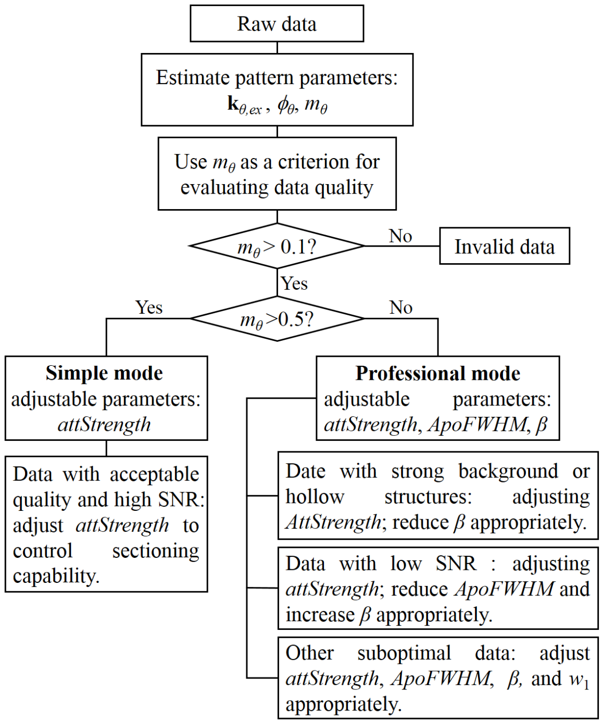

First, the pattern parameters, including wave vector $\mathbf{k}_{\theta,ex}$, initial phase ϕ_θ, and modulation depth m_θ from raw data, are determined by the amplitude normalized cross-correlation combined with a spectrum notch filter.

Then, the estimated modulation depth m_θ is used as a criterion for evaluating the quality of the raw data:

- If m_θ is less than 0.1, it indicates that no valid SIM pattern can be detected from the raw data. Treated as invalid SIM data and not used for further processing.

- If m_θ is greater than 0.5, the quality of the raw data is good.

  For data with such good quality, it is recommended to use the **Simple mode** of HiFi-SIM. In simple mode, the parameters w_1 = 1.2, β = 1.0 are fixed default values, and $ApoFWHM$ is automatically estimated by the excitation pattern spatial frequency and the detection objective cutoff frequency (**Supplementary Note 2**). Only $attStrength$ needs to be adjusted for controlling the sectioning capability (Fig. S20). In HiFi-SIM, the value of $attStrength$ is 0~1. When $attStrength$ = 0, OTF attenuation is turned off.

  The Simple mode can guarantee high-fidelity SR image without noticeable artifacts, which is suitable for most data and most ordinary users.

- If m_θ is greater than 0.1 but less than 0.5, the quality of the raw data is likely to be low, but it still contains many useful information.

For data with such low quality, it was recommended to be treated with special care by fairSIM because serious artifacts could appear. HiFi-SIM can still obtain high-quality SR-SIM images, but additional parameters, including $ApoFWHM$, β, and w_1, may need to be adjusted depending on the reason for the low modulation depth. We recommend the following range of values for these parameters: $ApoFWHM$ is 0.3–0.5; β is 0.2–2.0; w_1 is 0.9–2.5.

(1) For raw data with strong background or hollow structures: in addition to adjusting $attStrength$ to control the sectioning capability, appropriately reducing the “damping” factor β of the theoretical OTF is beneficial to remove the residual out-of-focus and related artifacts (Figs. 3d,e, 6a, S16 and S19c). In this case, the value range of β is 0.2 to 1.0.

(2) For raw data with low SNR, in addition to adjusting $attStrength$ to control the sectioning capability, appropriately reducing the parameter $ApoFWHM$ is beneficial to suppress artifacts related to high-frequency noise (Fig. S21). Moreover, appropriately increasing β can enhance the low-frequency and medium-frequency signals of the reconstructed spectrum, which is conducive to improving the reconstruction quality of data with low SNR (Figs. S22 and S23). In this case, the value range of β is 1.0 to 2.0.

(3) For suboptimal data that may be caused by low sample contrast, or poor optical alignment, in addition to adjusting $attStrength$ to control the sectioning capability, appropriately reduce both $ApoFWHM$ and β to obtain better quality reconstructed image.

In both **Simple mode** and **Professional mode**, OTF attenuation width $attWidth = 1.0$ and deconvolution wiener constant $w_2 = 1.0$ are fixed values by default. The value range of wiener constant w_1 is 0.9 to 2.5, which is 1.2 by default in this study. In addition, increasing w_1 appropriately helps to enhance the weak signal in the background area (Fig. S23f,g).

In summary, for most of the raw SIM data with acceptable quality, HiFi-SIM can reconstruct high-fidelity SR images with default parameters. For raw SIM data with suboptimal quality, users can easily reconstruct high-quality SR images by appropriately adjusting parameters according to the above guidelines. HiFi-SIM could make SR-SIM technology friendly to non-professional users and promote it to be widely used as a general daily imaging tool.

Supplementary References

1. Gustafsson, M.G. Surpassing the lateral resolution limit by a factor of two using structured illumination microscopy. *Journal of microscopy* **198**, 82–87 (2000).
2. Gustafsson, M.G. et al. Three-dimensional resolution doubling in wide-field fluorescence microscopy by structured illumination. *Biophysical journal* **94**, 4957–4970 (2008).
3. Wicker, K., Mandula, O., Best, G., Fiolka, R. & Heintzmann, R. Phase optimization for structured illumination microscopy. *Opt. Express* **21**, 2032–2049 (2013).
4. Wicker, K. Non-iterative determination of pattern phase in structured illumination microscopy using auto-correlations in Fourier space. *Opt. Express* **21**, 24692–24701 (2013).
5. Müller, M., Mönkemöller, V., Hennig, S., Hübner, W. & Huser, T. Open-source image reconstruction of super-resolution structured illumination microscopy data in ImageJ. *Nat. Commun.* **7**, 10980 (2016).
6. Huang X, et al. Fast, long-term, super-resolution imaging with Hessian structured illumination microscopy. *Nat. Biotechnol.* **36**, 451–459 (2018).
7. Demmerle, J. et al. Strategic and practical guidelines for successful structured illumination microscopy. *Nat. Protoc.* **12**, 988–1010 (2017).
8. Shaw, M., Zajiczek, L. & O'Holleran, K. High speed structured illumination microscopy in optically thick samples. *Methods* **88**, 11–19 (2015).
9. Ingerman, E. A., London, R. A. & Gustafsson, M. G. L. Signal, noise and resolution in linear and nonlinear structured-illumination microscopy. *J. Microsc.* **273**, 3–25 (2019).
10. Karras, C. et al. Successful optimization of reconstruction parameters in structured illumination microscopy – a practical guide. *Opt. Commun.* **436**, 69–75 (2019).
11. Young, L.J., Ströhl, F. & Kaminski, C.F. A guide to structured illumination TIRF microscopy at high speed with multiple colors. *J. Vis. Exp.* **111**, e53988 (2016).
12. Kraus F., et al. Quantitative 3D structured illumination microscopy of nuclear structures. *Nat. Protoc.* **12**, 1011–1028 (2017).
13. Křížek, P., Lukeš, T., Ovesný, M., Fliegel, K., & Hagen, G. M. SIMToolbox: A MATLAB Toolbox for Structured Illumination Fluorescence Microscopy. *Bioinformatics* **32**, 318–320 (2015).
14. Lal, A.; Shan, C. Y.; Xi, P. Structured Illumination Microscopy Image Reconstruction Algorithm. *IEEE J. Sel. Top. Quantum Electron*. **22**, 50–63 (2016).
15. Sahl, S.J. et al. Comment on "Extended-resolution structured illumination imaging of endocytic and cytoskeletal dynamics". *Science* **352**, 527 (2016).
16. O'Holleran, K., & Shaw, M. Polarization effects on contrast in structured illumination microscopy. *Opt. Lett.* **37**, 4603–4605 (2012).
17. Heintzmann, R., & Huser, T. Super-Resolution Structured Illumination Microscopy. *Chem. Rev.* **117**, 13890–13908 (2017).
18. Wu, Y. & Shroff, H. Faster, Sharper, and Deeper: Structured Illumination Microscopy for Biological Imaging. *Nat. Methods* **15**, 1011–1019 (2018).
19. Guo Y, Li D, Zhang S, et al. Visualizing Intracellular Organelle and Cytoskeletal Interactions at Nanoscale Resolution on Millisecond Timescales. *Cell* **175** 1430–1442 (2018).
20. Lukeš T., *et al.* Three-dimensional super-resolution structured illumination microscopy with maximum a posteriori probability image estimation. *Opt. Express* **22**,29805–29817 (2014).
21. O'Holleran, K. & Shaw, M. Optimized approaches for optical sectioning and resolution enhancement in 2D structured illumination microscopy. *Biomed. Opt. Express* **5**, 2580–2590 (2014).

22. Shabani, H., Doblas, A., Saavedra, G., Ortiga, E. S. & Preza, C. Improvement of two-dimensional structured illumination microscopy with an incoherent illumination pattern of tunable frequency. *Appl. Opt.* **57**, B92–B101 (2018).
23. Lai-Tim, Y., *et al.* Jointly super-resolved and optically sectioned Bayesian reconstruction method for structured illumination microscopy. *Opt. Express* **27**, 33251–33267 (2019).
24. Johnson, K.A. & Hagen, G. M. Artifact-free whole-slide imaging with structured illumination microscopy and Bayesian image reconstruction. *Gigascience* **9**, giaa035 (2020).
25. Hoffman, D. P. & Betzig, E. Tiled Reconstruction Improves Structured Illumination Microscopy. *BioRxiv* 895318 (2020) doi:10.1101/895318.
26. Fan, J. C. et al. A protocol for structured illumination microscopy with minimal reconstruction artifacts. *Biophys. Rep.* **5**, 80–90 (2019).
27. Perez, V., Chang, B.J. & Stelzer, E.H.K. Optimal 2D-SIM reconstruction by two filtering steps with Richardson-Lucy deconvolution. *Sci. Rep.* **6**, 37149 (2016).
28. Cao R., *et al.* Inverse matrix based phase estimation algorithm for structured illumination microscopy. *Biomed. Opt. Express* **9**, 5037–5051 (2018).
29. Sage, D. *et al.* DeconvolutionLab2: An Open-Source Software for Deconvolution Microscopy. *Methods* **115,** 28-41 (2017).
30. Li, D. *et al.* Extended-resolution structured illumination imaging of endocytic and cytoskeletal dynamics. *Science* **349**, aab3500 (2015).
31. Furuya, H., Eda, S. & Shimamura, T. Image restoration via Wiener filtering with improved noise estimation. *Proceedings of the 8th WSEAS international conference on Signal processing robotics and automation*, pp. 315-320 (2009).
32. Markwirth, A. *et al.* Video-rate multi-color structured illumination microscopy with simultaneous real-time reconstruction. *Nat. Commun.* **10**, 4315 (2019).
33. Chu, K. *et al.* Image reconstruction for structured-illumination microscopy with low signal level. *Opt. Express* **22**, 8687–8702 (2014).

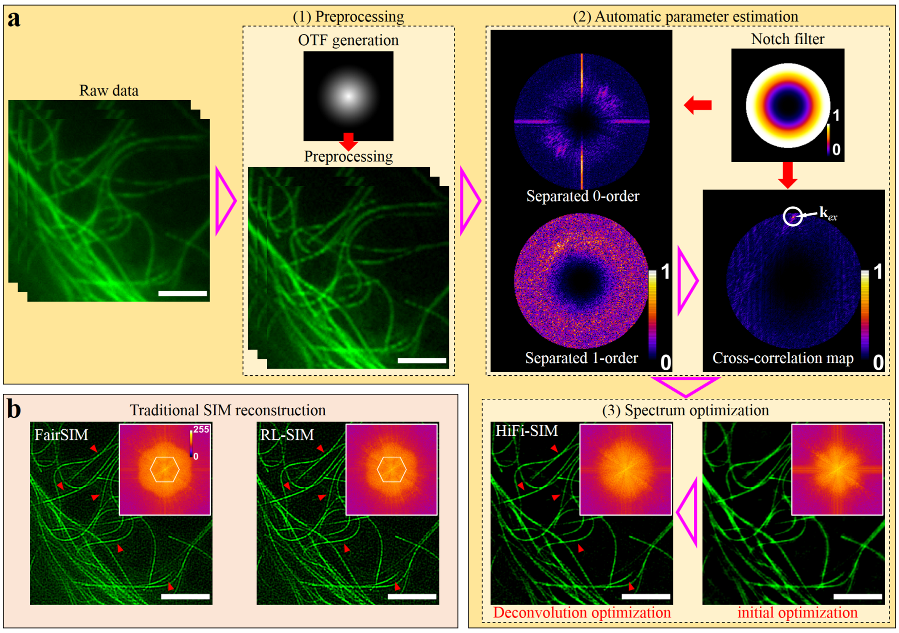

Figure S1 | Reconstruction flow of HiFi-SIM. a, Flow chart of HiFi-SIM, including preprocessing, automatic reconstruction parameter estimation, and spectrum optimization. **b,** Images of tubulin filaments, obtained by Wiener-SIM and Richardson-Lucy (RL) deconvolution SIM (RL-SIM)[27] in the open-source package fairSIM[5] (attenuation strength 0.99, FWHM 1.5 cycles/micron, Wiener parameter 0.1, RL iteration 5), for comparison. The right-upper corners show the corresponding spectrum. The TIRF-SIM data of tubulin filaments was from the fairSIM website. Scale bar: 3 μm.

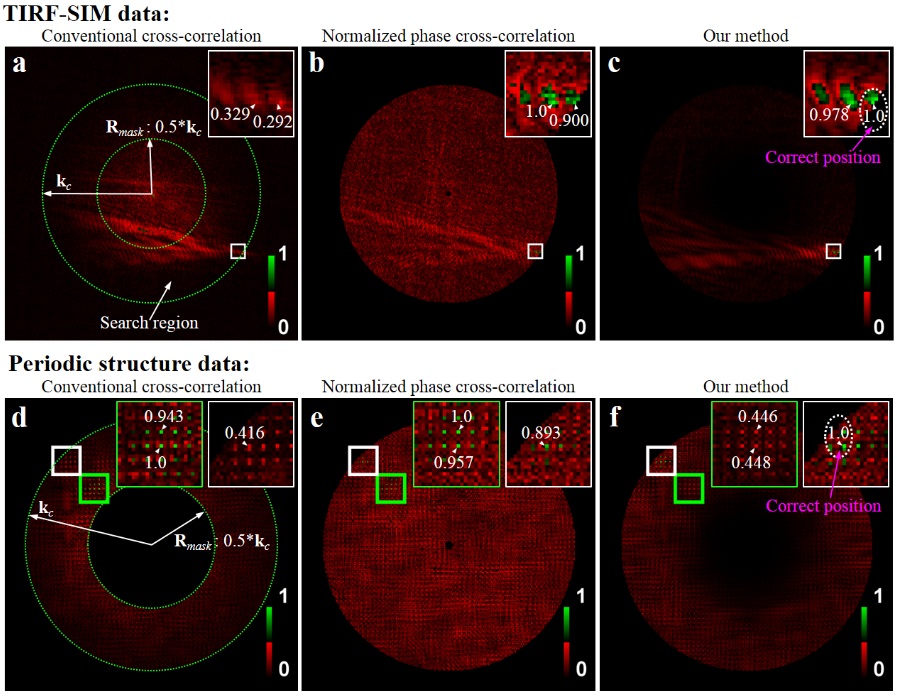

Figure S2 | Comparison of typical methods for estimating the pattern wave vectors. a-c, the cross-correlation maps obtained from a TIRF-SIM data set using the conventional cross-correlation method[5], normalized phase cross-correlation method[6,28], and our method. $\boldsymbol{k}_c$ is the cut-off frequency of wide-field imaging. **d-f**, the cross-correlation maps obtained from a periodic structure data using the conventional cross-correlation method, normalized phase cross-correlation method, and our method. By combination of amplitude normalized cross-correlation combined with the spectrum notch filter, our method can automatically find the correct peaks in the cross-correlation maps for the TIRF-SIM data and the periodic structure data.

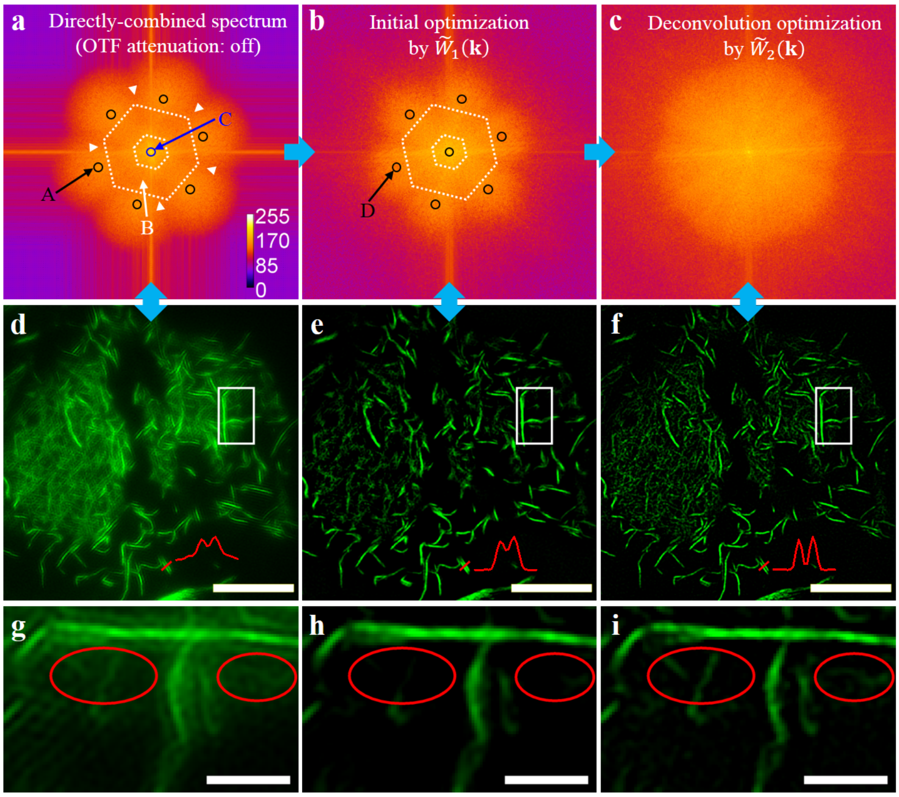

Figure S3 | Effects of the two-step spectrum optimization algorithm on the reconstructed spectrum and reconstructed images. Microfilaments in live COS7 cells expressing mEGFP-Lifeact were imaged by our home-built TIRF-SIM system with 488 nm excitation. **a,** directly-combined spectrum by summing all the shifted spectrum components, as Eq. (9) in Supplementary Note 1. **b,** reconstructed spectrum after initial optimization through $\widetilde{W}_1(\mathbf{k})$. **c,** reconstructed spectrum after further deconvolution optimization by $\widetilde{W}_2(\mathbf{k})$. **d-f**, reconstructed images corresponding to **a**-**c**. **g-i**, magnified images of the white box regions in (**d-f**). Note that the typical artifacts appearing in reconstructed image **(d)** are directly related to the abnormal features in the reconstructed spectrum **(a)**. Honeycomb artifacts correspond to multiple pairs of sharp residual background signal spectrum peaks in the center of the ±1-order shifted components (region A). Sidelobe artifacts correspond to the patchy features in the reconstructed spectrum (region B). The sectioning capability is limited by the spectrum peak of the residual out-of-focus background in the center of the reconstructed spectrum (region C). Application of $\widetilde{W}_1(\mathbf{k})$ can attenuate the signals in regions A and C, and correct the "kinked fluctuation" in region B. As a result, the honeycomb, sidelobe, and hammerstroke artifacts, as well as the residual defocused background are eliminated (**b,e,h**). However, the application of $\widetilde{W}_1(\mathbf{k})$ also causes region D to collapse downward, thereby causing the real sample structures to be attenuated or even lost (**h**). Application of $\widetilde{W}_2(\mathbf{k})$ not only improves the spatial resolution but also recovers the attenuated sample structures (red ellipses). Scale bar in (**a-c**), 5 µm; in (**g-i**) 1 µm.

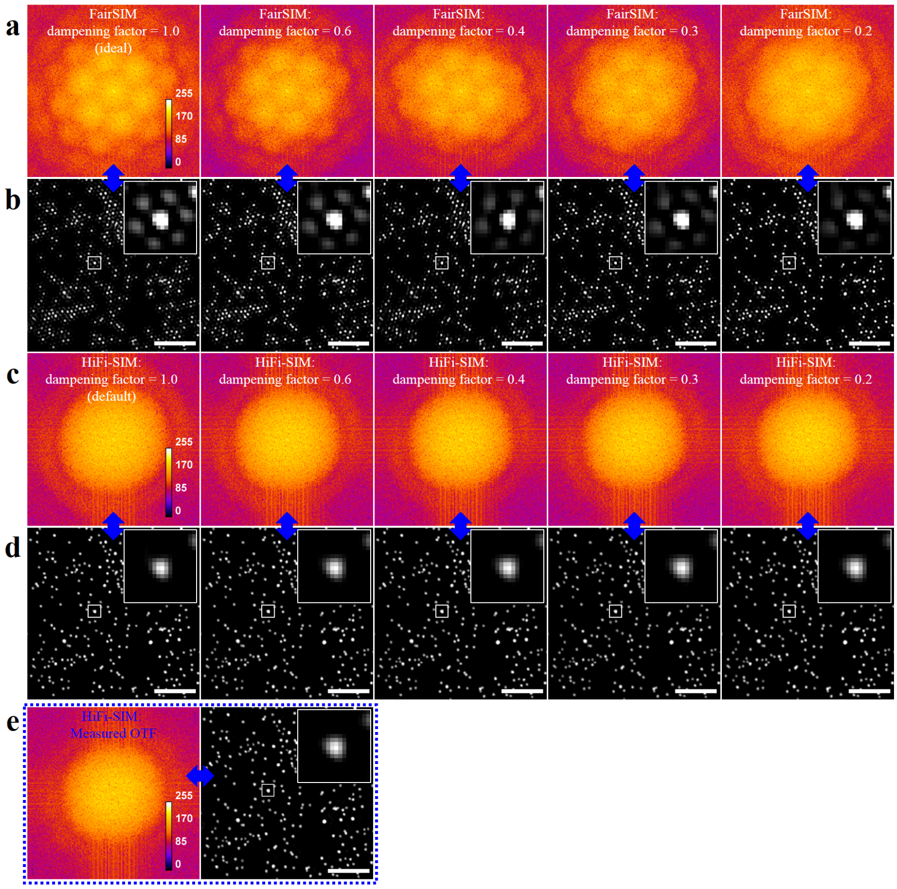

Figure S4 | Effects of different OTFs on the reconstructed results by fairSIM and HiFi-SIM. 100-nm fluorescent beads were imaged by our home-built TIRF-SIM system with 488 nm excitation. **a,** reconstructed spectrum by fairSIM using OTFs with “dampening” factor ranged from 0.2 to 1.0 (attenuation strength 1.0, FWHM 1.5 cycles/micron, Wiener parameter 0.1). **b,** corresponding reconstructed SR-SIM images. **c**, reconstructed spectrum by HiFi-SIM using the same OTFs as in **a**. **d**, corresponding reconstructed SR-SIM images. **e,** reconstructed spectrum by HiFi-SIM using the measured OTF, and the corresponding SR image. The right-upper corners show magnified individual beads in the white-box regions. Note that there are obvious patchy features in the reconstructed spectrum of fairSIM using OTFs with different dampening factor, resulting in obvious sidelobe artifacts in the reconstructed images. Gradually reducing the “dampening” factor is beneficial for correcting the patchy features of the reconstructed spectrum, thereby suppressing the sidelobe artifacts in the reconstructed images. By contrast, HiFi-SIM can effectively correct the patchy features that appear in the reconstructed spectrum of fairSIM to achieve high-quality SIM reconstruction with all the OTFs. This demonstrates that HiFi-SIM is more immune to reconstruction artifacts related to OTF mismatch than Wiener-SIM. Therefore, HiFi-SIM can employ the theoretical OTF generated based on imaging parameters for SIM reconstruction, avoiding the complicated calibration of the experimental OTF. Scale bar in (**b,d,e**) 2 μm.

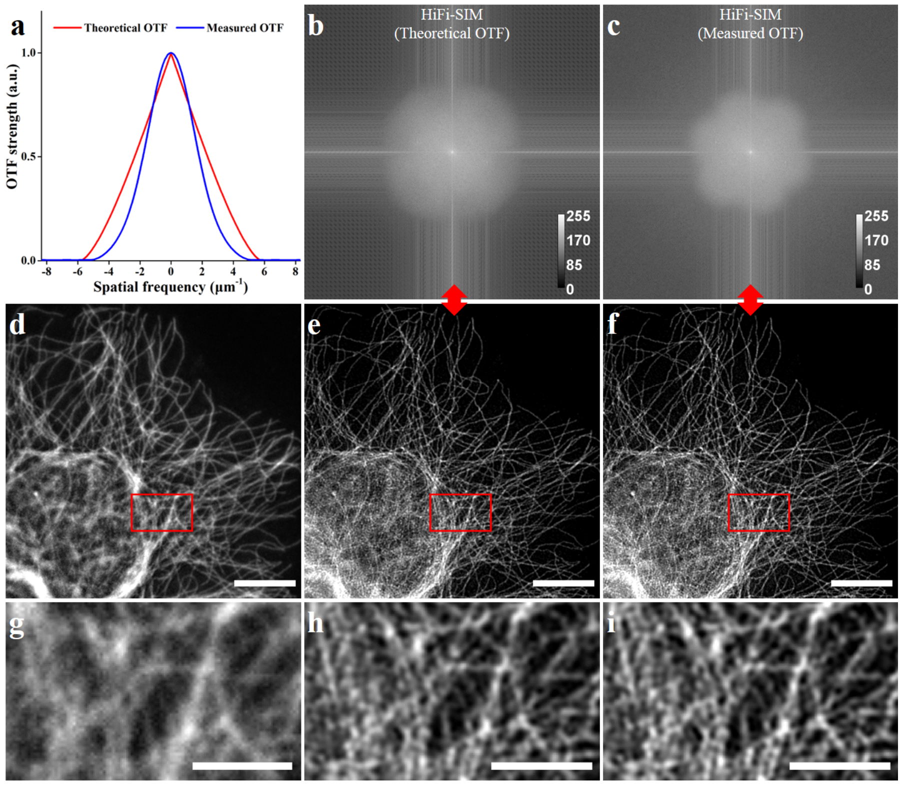

Figure S5 | Comparison of the reconstructed results of HiFi-SIM using the theoretical OTF and the accurately measured OTF, respectively. The test data of microtubules in COS7 cells and the measured OTF were from the OpenSIM algorithm[14]. **a,** Comparison of the theoretical OTF generated based on the imaging parameter (NA = 1.49, excitation wavelength = 488 nm, emission wavelength = 515 nm, calibration = 60 nm/pixel) and the accurately measured OTF. Of note, the cut-off frequency of the measured OTF seems to be smaller than that of the theoretical OTF, and the measured OTF has lower relative intensity in the medium- and high-frequency regions. This indicates that the theoretical OTF has obvious deviation with the actual imaging conditions. **b, c,** reconstructed spectrum of HiFi-SIM using the theoretical OTF and measured OTF, respectively. **d,** equivalent wide-field image. **e, f** SR images of HiFi-SIM using the theoretical OTF and the measured OTF, respectively. **g-i,** magnified images corresponding to the red box regions in (**d-f**). Note that the reconstructed image quality obtained by HiFi-SIM using the theoretical OTF is comparable to that obtained using the accurately measured OTF. This confirms that HiFi-SIM has a strong immunity to the reconstruction artifacts related to OTF mismatch. Therefore, HiFi-SIM uses a theoretical OTF by default, thus avoiding complex and professional OTF calibration. Scale bar in (**d-f**), 6 μm; in (**g-i**), 2 μm.

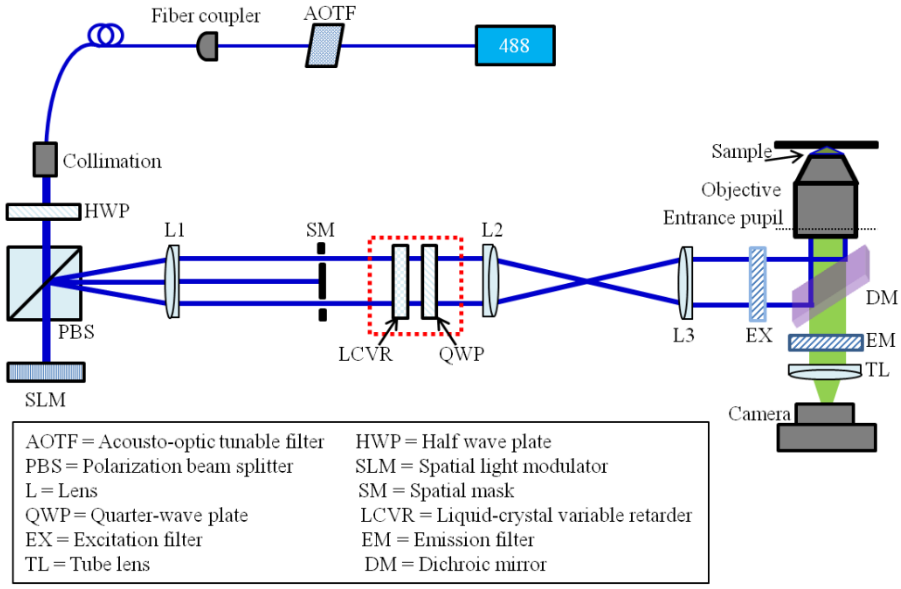

Figure S6 | Layout of our home-built 2D-SIM system. Our 2D-SIM setup was constructed around a commercial inverted fluorescence microscope (IX83, Olympus, Japan). An exciting light beam of 488 nm (Genesis MX488-500 STM, Coherent, USA) is coupled into a single-mode polarization-maintaining optical fiber after strobed by an acoustic-optic tunable filter (AOTFnC-VIS, AA Opto-Electronic, France). The emergent laser beam is collimated to a diameter of 15 mm by using a super apochromatic objective (TL2X-SAP, Thorlabs Inc., USA) and then transmitted through an achromatic half-wave plate (AHWP10M-600, Thorlabs Inc., USA) and polarization beam splitter (PBS) (PBS251, Thorlabs Inc., USA). Then the beam is vertically projected onto a ferroelectric liquid-crystal spatial light modulator (SLM, SXGA-3DM, Fourth Dimension Displays, UK). Diffracted beams pass through an optical 4*f* system consisting of two self-designed achromatic doublets, L1 (f1 = 300 mm) and L2 (f2 = 150 mm), and enters an IX83 microscope. At the focal plane of lens L1, a spatial mask (SM) consisting of six symmetrical 0.2 mm diameter pinholes is used to filter out ±1-order diffraction beams. A liquid-crystal variable retarder (LVR-200-VIS-TSC-1L, Meadowlark Optics, USA) and a quarter wave plate (AQWP05M-600, Thorlabs Inc., USA) was employed to modulate the polarization state of the filted ± 1 order diffraction beams. The two beams enter an Oil immersion objective (NA = 1.49, UAPON 100× OTIRF, Olympus, Japan) from two side to form high contrast sinusoidal pattern at the focal plane. The period of the pattern could be adjusted by changing the loaded image at SLM for TIRF-SIM or convention SIM with incident beam angle smaller than the critical angle of TIRF. A scientific sCMOS camera (ORCA Flash 4.0 V2, Hamamatsu Photonics) was used to capture fluorescent images.

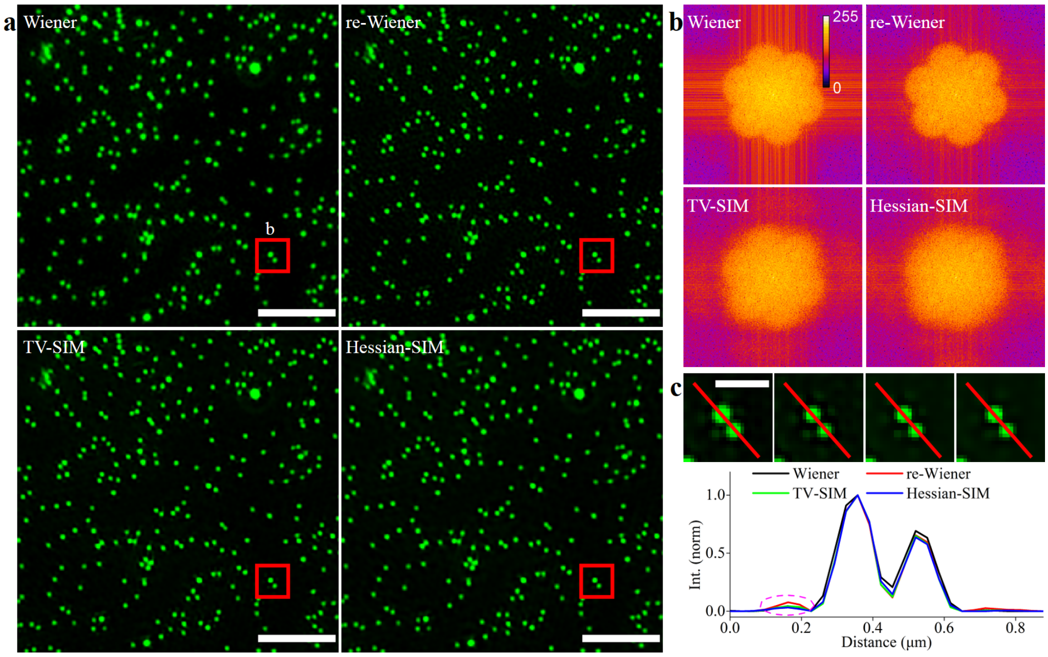

Figure S7 | Comparison of the reconstructed results of different algorithms using the accurately measured OTF. Raw data is the same as in Fig. 2. **a**, SR images reconstructed by the traditional Wiener-SIM we implemented, and the Wiener-SIM (called 're-Wiener'), TV-SIM and Hessian-SIM implemented in [6], using the accurately measured OTF. **b**, Reconstructed spectrum corresponding to **a**. **c**, Magnified images of the red-box regions in **a**, and the line profiles along the beads in **c**. Compared the reconstructed spectrum using theoretical OTFs (Fig. 2), and found that almost no obvious patchy features are found in the reconstructed spectrum by using measured OTF. Therefore, artifacts in the reconstructed image are significantly reduced. Comparing Fig. 2b and Fig. S7c, the intensity of residual sidelobe artifacts is significantly reduced, but still exists. Scale bar in **a**, 2 µm; in **c**, 0.5 µm.

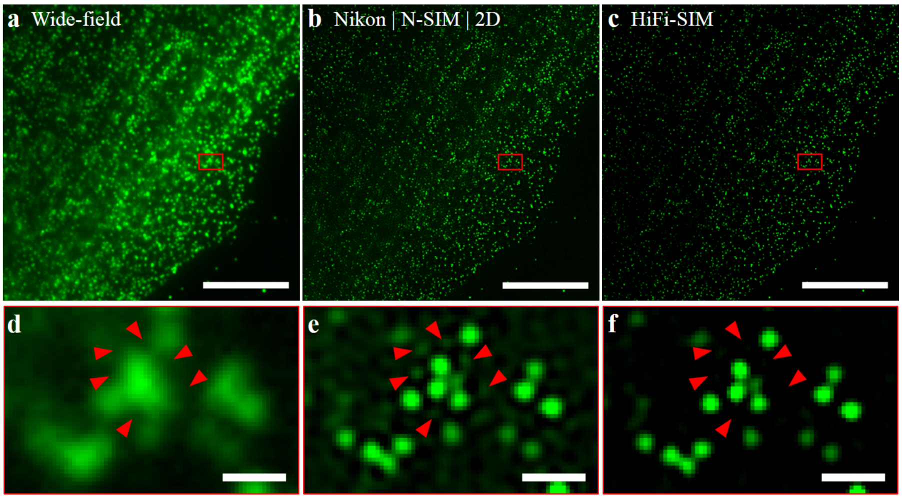

Figure S8 | Performance of HiFi-SIM on reconstructing SIM image of caveolae in live cells. Caveolae in live U2OS cells expressing mEmerald-Caveolin were imaged by a Nikon N-SIM under 2D-SIM mode with 488 nm excitation. **a**, equivalent wide-field image. **b**, SR image reconstructed by Nikon NIS-Elements using the calibrated OTF. **c**, SR image reconstructed by HiFi-SIM using the theoretical OTF. **d-f**, magnified images corresponding to the red box regions in (**a-c**). Reconstruction by Wiener-SIM of microsphere-like structures such as caveolae often yields snowflake-like sidelobe artifacts symmetrically around them (**b, e**). Because these snowflake-like sidelobes appeared as dots, they are likely to be misinterpreted as real. Moreover, if two caveolae close, their sidelobes can be superimposed together which makes them harder to distinguish. With these snowflake-like sidelobe artifacts mostly eliminated, HiFi-SIM considerably improves the quality and fidelity of SR images (**c, f**). Scale bar in (**a-c**) 3 µm; in (**d-f**), 0.5 µm.

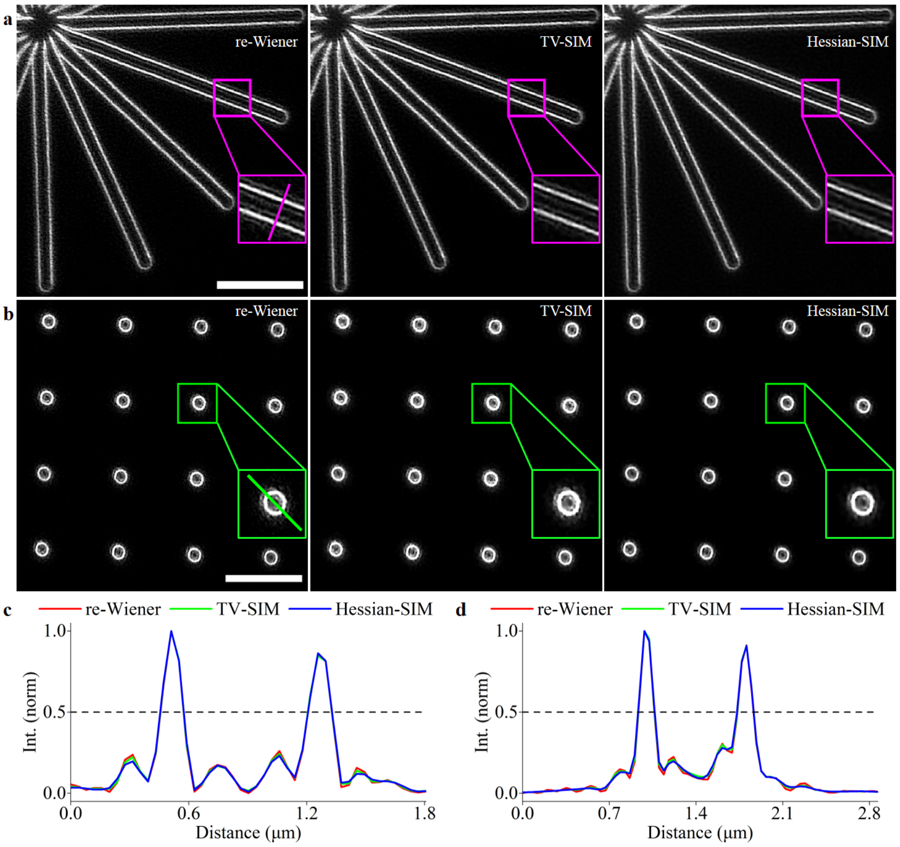

Figure S9 | Performance of re-Wiener, TV-SIM, and Hessian-SIM on reconstructing SR images of "Start" and "2D matrix of rings" patterns in Argolight slide. Raw data is the same as in Fig. 3. **a, b**, SR images of "line" and "ring" structures reconstructed by re-Wiener, TV-SIM, and Hessian-SIM implemented in [6]. **c**, Intensity profiles along the magenta line in **a**. **d**, Intensity profiles along the green line in **b**. Here, the three algorithms all use the same OTF as used in fairSIM (Fig. 3), and use the built-in notch filters to suppress pattern artifacts (a_0 = 0.05, v = 1.2). Similar to the reconstruction results of fairSIM, and GE SoftWoRx in Fig. 3, there are obvious sidelobe artifacts and residual background in the images of re-Wiener, TV-SIM, and Hessian-SIM. Of note, TV and Hessian deconvolution cannot effectively eliminate the sidelobe artifacts generated in Wiener-SIM step. Scale bar in **a, b,** 5 μm.

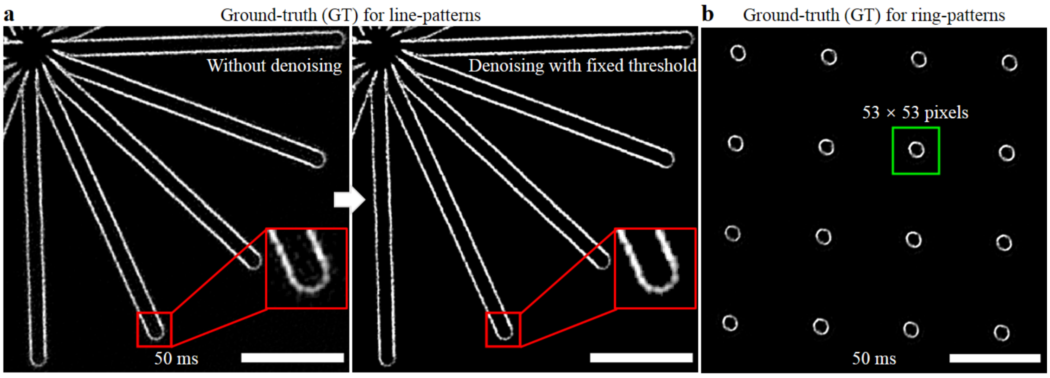

Figure S10 | Ground-truth (GT) images for the "Start" and "2D matrix of rings" patterns in Argo-SIM slide. a, GT image of the "start" patterns (line structures). Left: SR image reconstructed by HiFi-SIM from high signal-to-noise ratio (SNR) data with 50 ms exposure time. Right: SR image denoised by a fixed threshold is used as the GT image. **b,** GT image of the "2D matrix of rings" patterns (ring structures) was obtained by using HiFi-SIM to reconstruct high SNR contrast data with 50 ms exposure. The green-box region corresponds to the magnified image of Fig. 3e. Scale bar in (**a, b**), 5 µm.

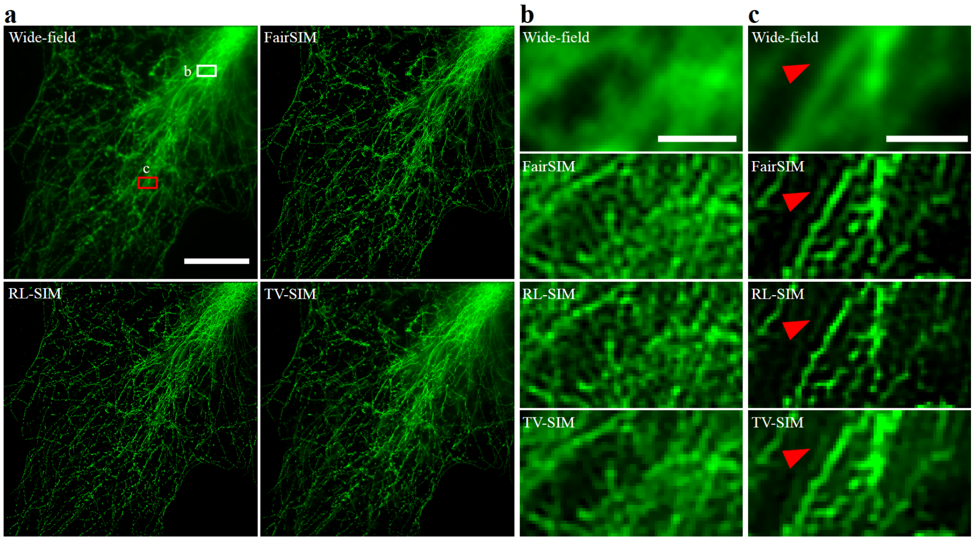

Figure S11 | Reconstruction of raw data with high SNR and large dynamic range. Raw data is the same as in Fig. 4d. **a**, the equivalent wide-field image is shown in the upper left corner, and the SR images reconstructed by fairSIM (attenuation strength 0.999, FWHM 1.5 cycles/micron, Wiener parameter 0.1), RL-SIM[27] in fairSIM (attenuation strength 0.999, FWHM 1.5 cycles/micron, Wiener parameter 0.1, RL iteration 5), and TV-SIM implemented in [6], respectively. Here, TV-SIM used the same OTF as used in fairSIM. **b, c,** Magnified images of the equivalent wide-field, and the corresponding SR images in the red-box and white-box regions in **a**. Note that high SNR contrast raw data to ensure that fairSIM, RL-SIM, and TV-SIM can reconstruct good SR images. However, some local regions of these reconstructed images contain obvious sidelobe and non-continuous artifacts, and residual out-of-focus background. The SR images reconstructed by GE SoftWoRx and Hessian-SIM also contain these artifacts (Fig. 4d-f). In contrast, these artifacts are well suppressed by HiFi-SIM (Fig. 4d-f). Scale bar in **a**, 10 µm; in (**b, c**), 1 µm.

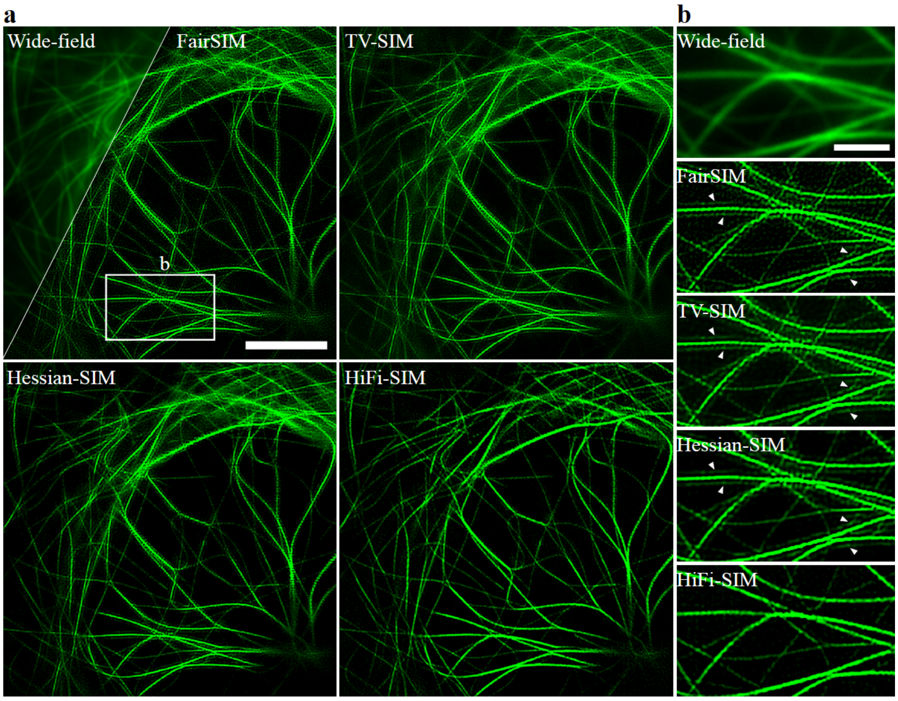

Figure S12 | Comparison of sidelobe artifacts in reconstructed images of fairSIM, TV-SIM, Hessian-SIM, and HiFi-SIM. Microtubules in live COS-7 cells expressing mEGFP-Lifeact were imaged by our home-built TIRF-SIM system with 488 nm excitation. **a,** the equivalent wide-field image is shown in the left triangle, and the SR images were reconstructed using fairSIM, TV-SIM, Hessian-SIM, and HiFi-SIM, respectively. Among them, the reconstruction results of TV-SIM and Hessian-SIM were obtained by further implementing TV- and Hessian-denoising procedures[6] on the result of fairSIM. **b,** Magnified images of the equivalent wide-field, and the corresponding SR images in the white-box region in **a**. Note that there are obvious sidelobe, and discontinuous hammerstroke artifacts in the reconstructed image of fairSIM. With TV- and Hessian-denoising, the discontinuous hammerstroke artifacts in the background region were well suppressed, but the sidelobe artifacts cannot be eliminated. In particular, the sidelobe artifacts in TV-SIM and Hessian-SIM become more continuous, which increases the risk of being misjudged as real structures. Moreover, some weak structures with intensity close to the background level were lost in the reconstructed images of fairSIM, TV-SIM and Hessian-SIM. In contrast, the reconstructed image of HiFi-SIM contains few sidelobe and hammerstroke artifacts, and retains the weak structures in the background area better. Scale bar in **a**, 6 µm; in **b**, 2 µm.

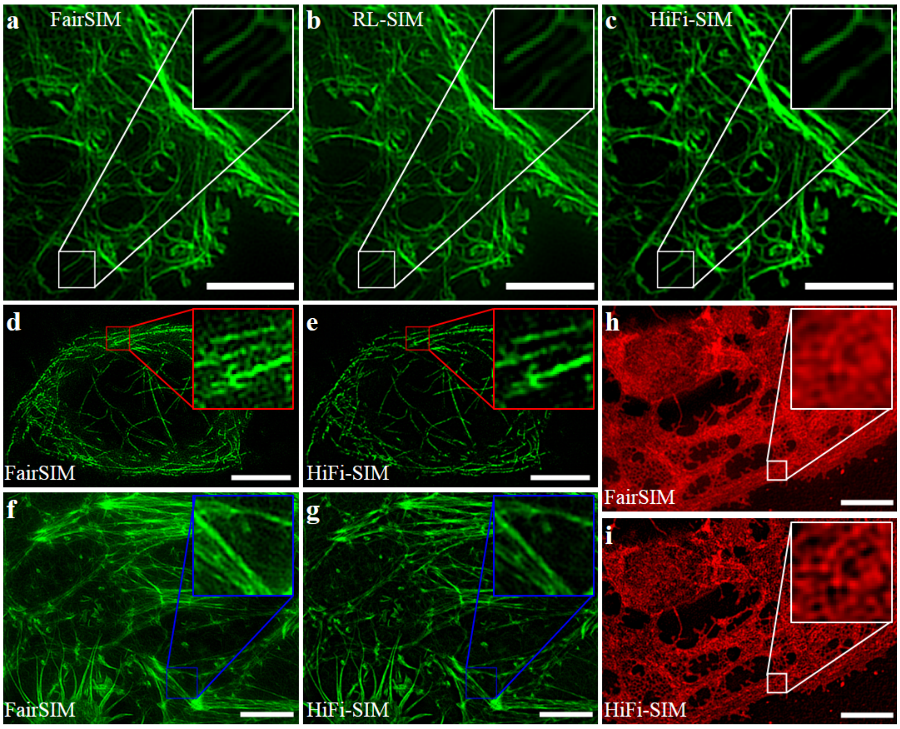

Figure S13 | Comparison of fairSIM and HiFi-SIM on reconstructing open-source data sets. a-c, SR images of actin filaments in liver sinusoidal endothelial cells (LSECs) (**Sample 1**) were reconstructed by Wiener-SIM and RL-SIM in fairSIM, and HiFi-SIM. Note that there are obvious sidelobe artifacts and residual out-of-focus background in **a** and **b**. HiFi-SIM can effectively suppress these artifacts and remove the residual background, so as to obtain SR image with higher fidelity (**c**). **d, e**, SR images of tubulin network in U2OS cells (**Sample 2**) were reconstructed by fairSIM and HiFi-SIM. Noted that the strong background fluorescence in the raw data caused obvious discontinuous artifacts in **d**. HiFi-SIM generated a "cleaner" SR image with individual microtubules much easier to be identified (**e**). **f, g**, SR images of actin filaments in U2OS cells (**Sample 3**) were reconstructed by fairSIM and HiFi-SIM. Note that HiFi-SIM effectively removed the sidelobe and hammerstroke artifacts, as well as the residual background contained in **f**. **h, i** SR images of plasma membrane in LSECs (**Sample 4**) were reconstructed by fairSIM and HiFi-SIM. Note that HiFi-SIM allows for clearer identification of the cellular fenestration (nano-sized membrane pores)[5]. All test data sets (**Sample 1**: OMX_LSEC_Actin_525nm.tif; **Sample 2**: OMX_U2OS_Tubulin_525nm.tif; **Sample 3**: OMX_U2OS_Actin_525nm.tif; **Sample 4**: OMX_LSEC_Membrane_680nm.tif) were downloaded from the fairSIM website, where detailed data information and imaging parameters can be found. Scale bar in (**a-c**), 4 μm; in (**d-g**), 5 μm; in (**h, i**), 4 μm.

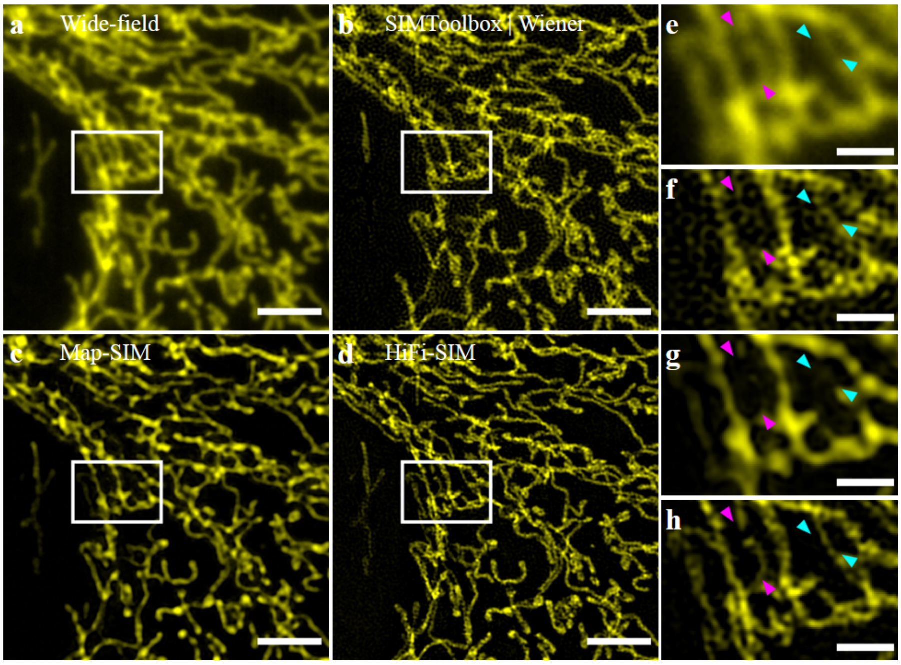

Figure S14 | Comparison of SIMToolbox and HiFi-SIM on reconstructing SIM images from open-source data. Mitochondrial data acquired by a Zeiss Elyra (**Sample**: **Mito_Zeiss.tif**) was downloaded from the SIMToolbox website[13], where data information and imaging parameters can be found. **a**, equivalent wide-field image. **b**, SR image reconstructed by Wiener-SIM in SIMToolbox. **c**, SR image reconstructed by MAP-SIM in SIMToolbox. **d**, SR image reconstructed by HiFi-SIM. **e-h**, magnified images of the white box regions in (**a-d**). All reconstruction parameters of Wiener-SIM and MAP-SIM are the same as the paper. Of note, there is a tradeoff between eliminating artifacts and retaining the real sample structures in SIM reconstruction (**Supplementary Note 1**). Here, the SR image of Wiener-SIM contains serious artifacts, and some sample structures visible in the equivalent wide-field image were lost (**b, f**). In the SR image of Map-SIM, the fine structures of mitochondria were also lost (**c, g**). In contrast, HiFi-SIM balances the tradeoff between eliminating artifacts and preserving sample structures well, and the reconstructed SR image has better quality and higher fidelity (**d, h**). Scale bar in (**a-d**), 3 μm; in (**e-h**), 1 μm.

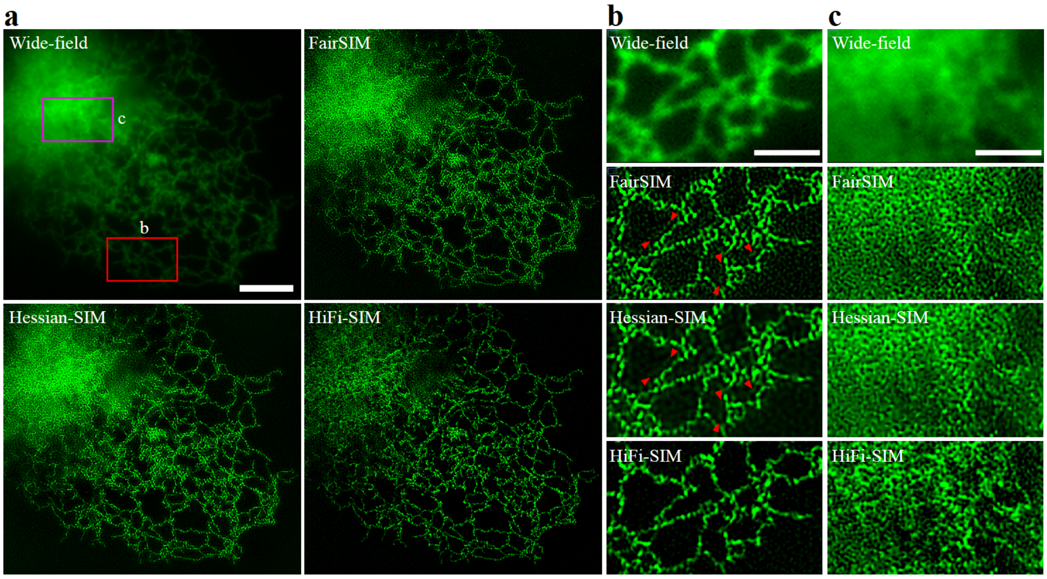

Figure S15 | Performance of HiFi-SIM on reconstructing SIM image of endoplasmic reticulum with strong background. Endoplasmic reticulum in fixed U2OS cells expressing mEmerald-ER3 were imaged by our home-built 2D-SIM system with 488 nm excitation. **a**, Equivalent wide-field image is shown in the upper left corner, and the SR images were reconstructed using fairSIM, Hessian-SIM, and HiFi-SIM, respectively. **b, c**, Magnified images of the equivalent wide-field, and the corresponding SR images in the red-box and magenta-box regions in **a**. Note that SR image reconstructed by fairSIM (attenuation strength 0.999, FWHM 1.5 cycles/micron, Wiener parameter 0.1) exhibit both sidelobe and hammerstroke artifacts. SR image obtained by further performing the Hessian denoising procedure on the result of fairSIM shows less hammerstroke artifacts but sidelobe artifacts enhanced. SR image reconstructed by HiFi-SIM shows no observable sidelobe (**b**), and less hammerstroke artifacts and residual background fluorescence (**c**). Scale bar in **a**, 5 µm; in (**b, c**), 2 µm.

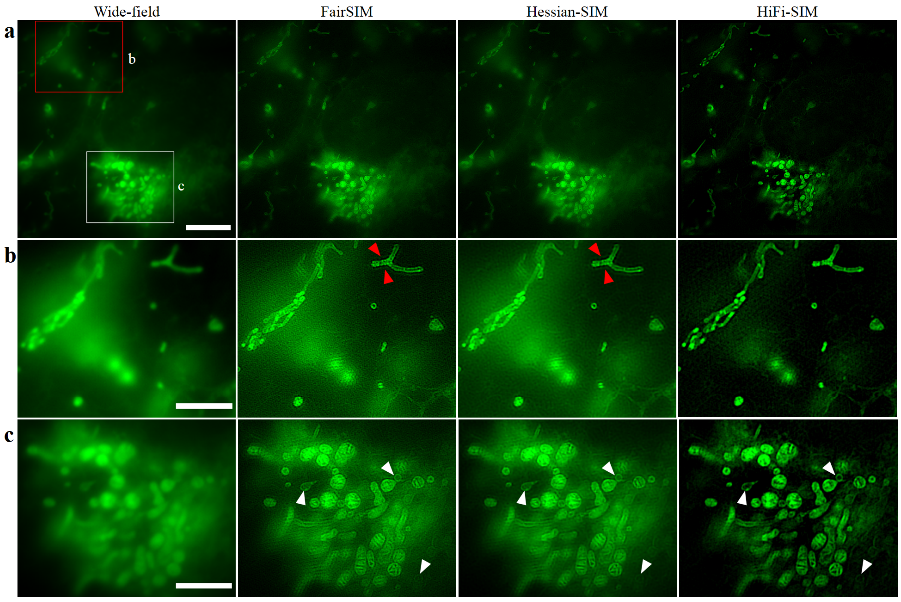

Figure S16 | Performance of HiFi-SIM on reconstructing SIM image of mitochondria with low modulation depth and strong background. Raw data of mitochondrial in live U2OS cells was downloaded from reference [32], where data information and imaging parameters can be found. **a,** Equivalent wide-field image is shown in the left corner, and the SR images were reconstructed by fairSIM, Hessian-SIM, and HiFi-SIM, respectively. Among them, the results of fairSIM and Hessian-SIM were downloaded from [32]. **b,** Magnified images of the red-box region in **a**. **c,** Magnified images of the white-box region in **a**. Note that there are sidelobe artifacts around some mitochondria in the reconstructed images of fairSIM and Hessian-SIM (**b**). In particular, Hessian-SIM can suppress the discontinuous artifacts in the image reconstructed by fairSIM, but the discontinuous segmented artifacts become more continuous. In addition, the surrounding and center of some larger mitochondria were filled with residual background fluorescence (**c**). Instead, HiFi-SIM suppresses the sidelobe artifacts and removes the residual background fluorescence, so that the corresponding mitochondria can be resolved into hollow structures. Scale bar in **a**, 8 μm; in (**b, c**), 4 μm.

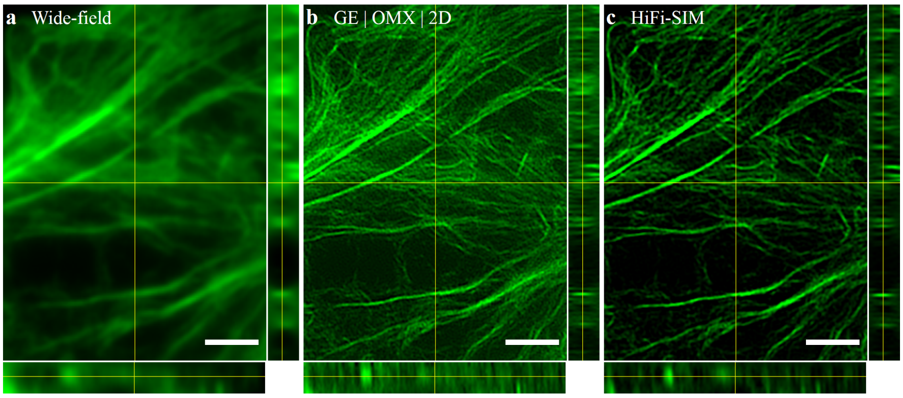

Figure S17 | Performance of HiFi-SIM on reconstructing 3D stack SIM data of thick samples. Microfilaments in fixed U2OS cells expressing mEGFP-Lifeact were imaged by a GE DeltaVision OMX SR under two-beam interference SIM mode in a 3D stack (9 layers, axial step 125 nm). **a**, Orthogonal view of equivalent wide-field image. **b**, Orthogonal view of 3D SR images reconstructed by 2D reconstruction of SoftWoRx (Wiener parameter 0.005) plane-by-plane. **c**, Orthogonal view of 3D SR images reconstructed by 2D reconstruction of HiFi-SIM plane-by-plane. With better sectioning capability, HiFi-SIM reconstructed SR images with higher contrast. Scale bar: 2 µm.

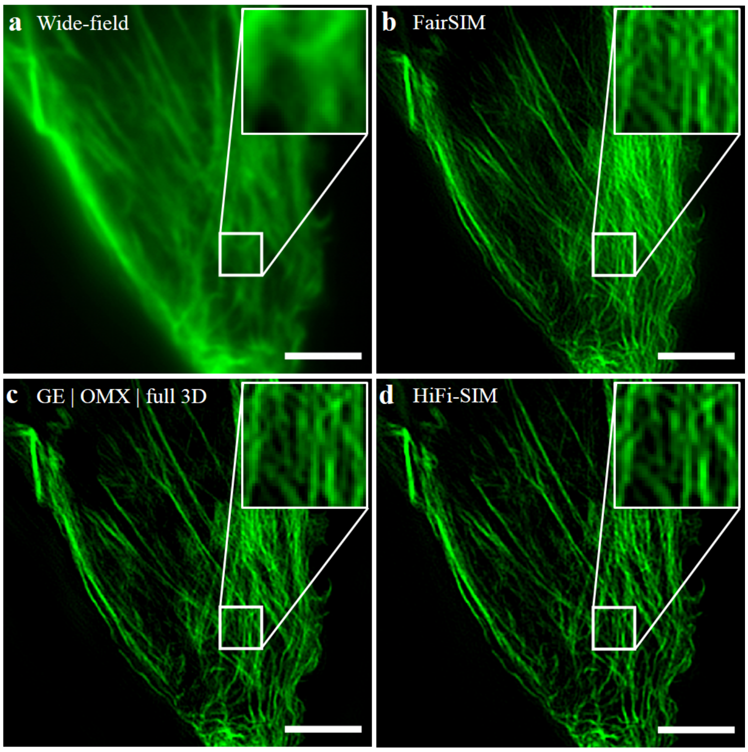

Figure S18 | Performance of HiFi-SIM on reconstructing single slice 3D-SIM raw data. Microfilaments in fixed U2OS cells expressing mEGFP-Lifeact were imaged by a GE DeltaVision OMX SR under 3D-SIM mode. **a**, equivalent wide-field image of a typical single slice. **b**, SR image of the same slice reconstructed by fairSIM (strength 1.0, FWHM 1.5 cycles/micron, Wiener parameter 0.1). **c**, the slice from 3D SR reconstructed by SoftWoRx (Wiener parameter 0.005). **d**, SR image of the same slice reconstruction by HiFi-SIM. Although HiFi-SIM is a 2D-SIM reconstruction, its sectioning capability effectively removes the residual out-of-focus background, reconstructed SR image with quality comparable to that of the same layer in the full 3D reconstruction by SoftWoRx. Scale bar: 5 μm.

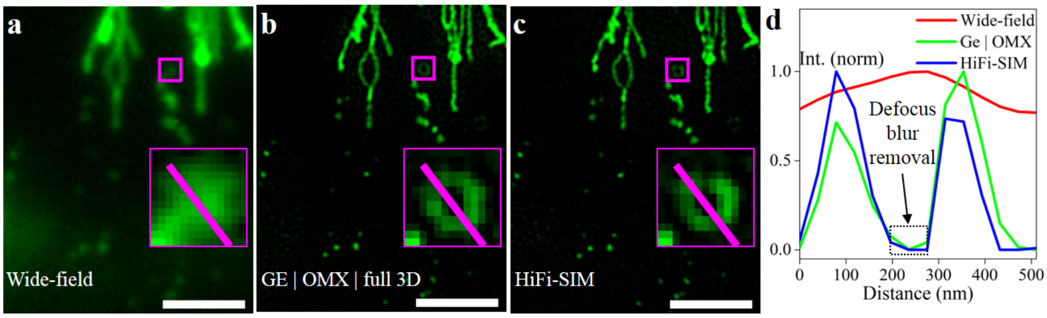

Figure S19 | Performance of HiFi-SIM on reconstructing SIM image of vesicles in live cells. Vesicles in live U2OS cells expressing CD63-EGFP was imaged by a GE DeltaVision OMX SR in three-beam 3D-SIM mode. **a,** equivalent wide-field image of one typical slice from 3D data sets. **b**, the same slice from full 3D reconstruction by SoftWORX (Wiener parameter 0.005). **c**, the same slice reconstruction by HiFi-SIM. **d**, intensity profiles along the magenta lines in (**a-c**). Ideally, vesicles with a size smaller or larger than SIM resolution should be resolved as beads or hollow rings, respectively. However, in the reconstructed images of fairSIM and Hessian-SIM, both the center and periphery of large vesicles exhibit significant residual out-of-focus background and related artifacts (Fig. 6). In contrast, HiFi-SIM can resolve them as hollow rings (**c**), similar to the results by 3D-SIM(**b**). Scale bars in (**a-c**), 2 µm.

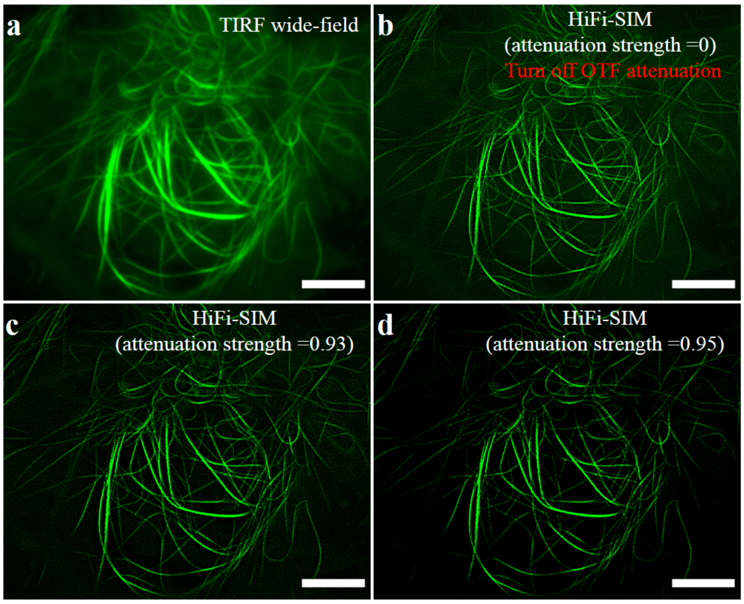

Figure S20 | Effect of OTF attenuation strength on the reconstructed image quality of HiFi-SIM. Microtubules in live COS-7 cells expressing mEmerald-Tubulin-N-18 were imaged by our home-built TIRF-SIM system with 488 nm excitation. **a**, equivalent wide-field image. **b**, Reconstructed image by HiFi-SIM without OTF attenuation (attenuation strength = 0). **c**, Reconstructed image by HiFi-SIM with attenuation strength = 0.93. **d**, Reconstructed image by HiFi-SIM with attenuation strength = 0.95. Note that OTF attenuation can achieve better optical sectioning capability, and appropriately adjusting the attenuation strength is beneficial to suppress the residual artifacts in the background region. Scale bar: 5 μm.

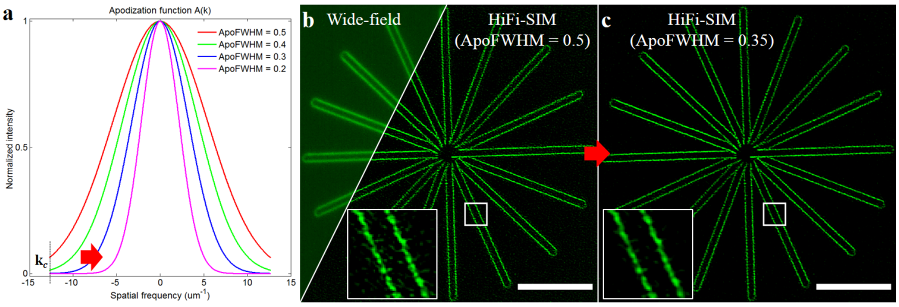

Figure S21 | Effect of parameter *ApoFWHM* on the reconstructed image quality of HiFi-SIM. The ring patterns in Argolight slide was imaged by a DeltaVision OMX SR, excited at 488 nm wavelength with 1 ms exposure time, resulting very low SNR. **a**, a Gaussian-shape function was employed as the apodization function in the reconstruction of HiFi-SIM. By appropriately adjusting the full width at half maximum (FWHM) of the apodization function (parameter $ApoFWHM$), high-frequency noise can be effectively attenuated, thereby suppressing artifacts related to high-frequency (**b, c**). We recommend setting $ApoFWHM$ between 0.3 and 0.5. Note that the smaller $ApoFWHM$ may sacrifice lateral resolution. Scale bar in (**b, c**), 8 μm.

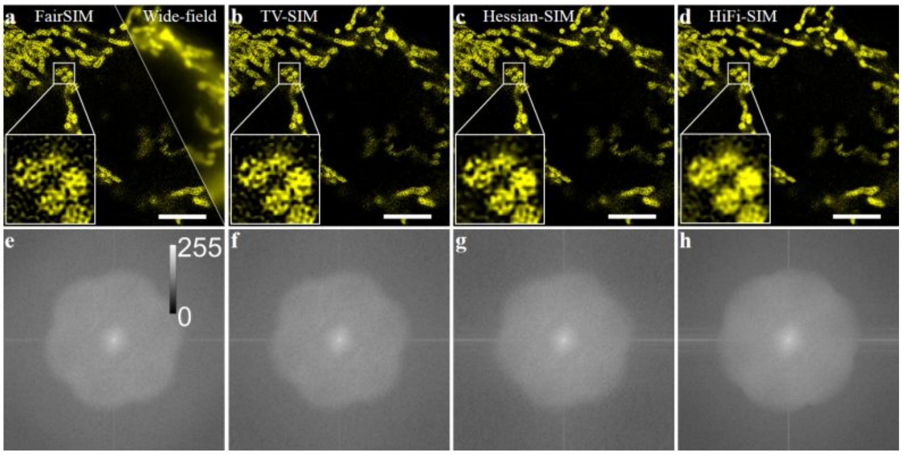

Figure S22 | Performance of HiFi-SIM on reconstructing raw data with low signal-to-noise ratio. Mitochondrial SIM data with low SNR from the fairSIM website was employed for testing. **a**, SR image reconstructed by fairSIM (attenuation strength 1.0, FWHM 1.5 cycles/micron, Wiener parameter 0.1). **b, c** SR images obtained by further denoising the result of fairSIM using the TV and Hessian denoising procedures in [6]. **d**, SR image reconstructed by HiFi-SIM ($attStrength$ 0.9, $ApoFWHM$ 0.35, β 2.0, w_1 2.5). Note that HiFi-SIM can effectively suppress artifacts related to high-frequency noise by adjusting the FWHM of the apodization function, thereby reconstructing SR images with high quality. Scale bar in (**b, c**), 8 μm; in (**d-g**), 5 μm.

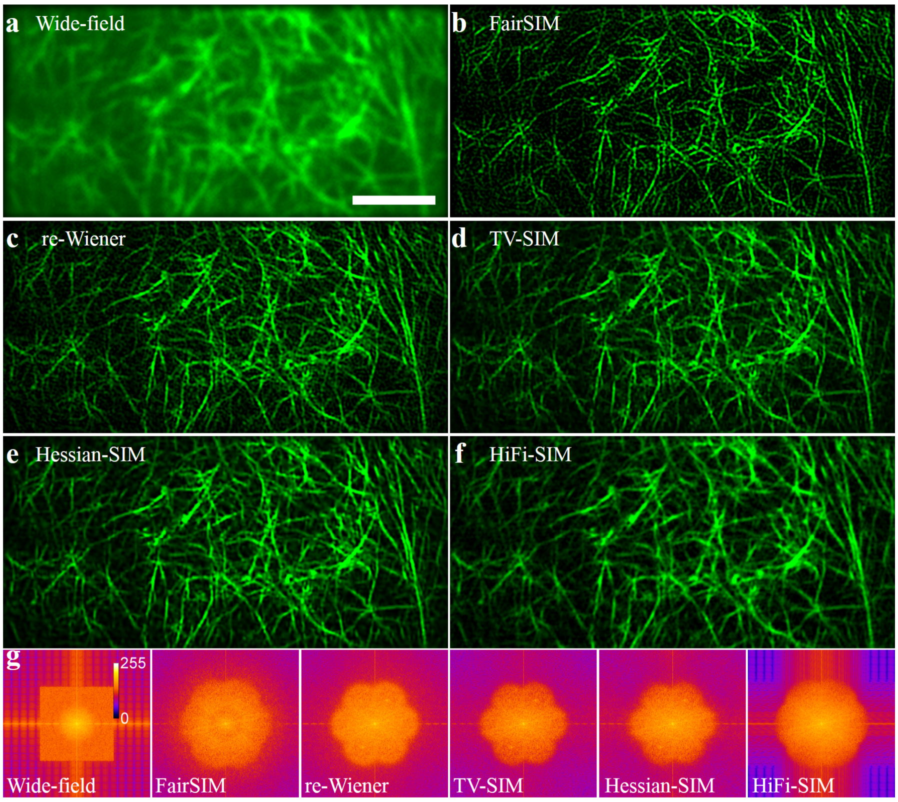

Figure S23 | Performance of HiFi-SIM on reconstructing raw data with sub-ms exposures. The open source data of densely packed actin filaments in human umbilical vein endothelial cells (HUVECs) with 0.5 ms exposures from reference [6] were employed for testing. **a**, equivalent wide-field image. **b**, SR image reconstructed by fairSIM (attenuation strength 1.0, FWHM 1.5 cycles/micron, Wiener parameter 0.1). **c-e**, SR images reconstructed by re-Wiener, TV-SIM, and Hessian-SIM in the open source software package in [6], and the parameter settings are the same as in the paper. **f**, SR images reconstructed by HiFi-SIM ($attStrength$ 0.95, $ApoFWHM$ 0.45, β 2.0, w_1 1.2). **g**, Spectrum of **a-f**. Although the SNR contrast of the raw data is low, the quality of SR image reconstructed by HiFi-SIM is better than that of fairSIM, re-Wiener and TV-SIM, and Hessian-SIM. Scale bar: 3 μm.

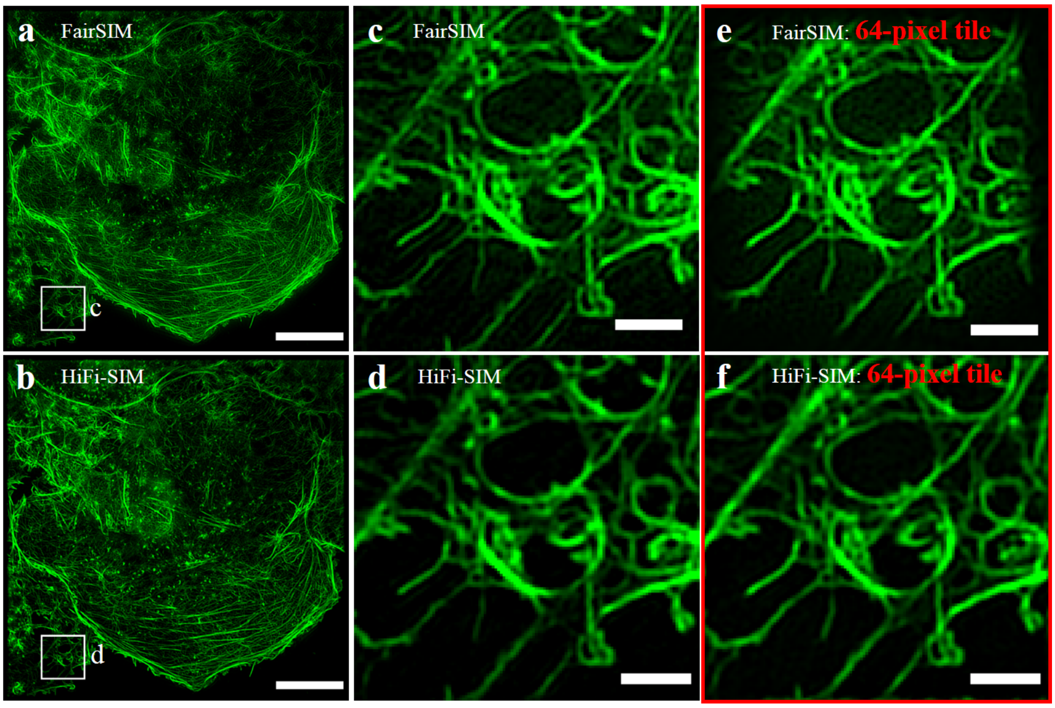

Figure S24 | Comparison of global reconstruction of Wiener-SIM and HiFi-SIM with local reconstruction of Tiled-SIM[25]. Raw SIM data is the same as in Fig. S13a-c. **a, b,** SR images were reconstructed by fairSIM and HiFi-SIM, respectively. **c, d,** Magnified images of the white-box regions in (**a, b**). **e, f,** SR images of a single sub-block in 64-pixel Tiled-SIM, which were reconstructed by fairSIM and HiFi-SIM, respectively. It is worth noting that fairSIM and HiFi-SIM reconstruct the whole field of view (512 × 512 pixels) at a single time, while Tiled-SIM reconstruct the local field of view (64 × 64 pixels). Compare **c** and **e**, 64-pixel tile reconstruction was helpful to suppress artifacts, such as sidelobe artifacts, in traditional Wiener-SIM reconstruction, and enhance weak signals in background area. However, all the sub-blocks of the initial Tiled-SIM are reconstructed by traditional Wiener-SIM. Therefore, Tiled-SIM reconstruction inevitably faces the same problems as the traditional Wiener-SIM. Here, we recommend using HiFi-SIM instead of the traditional Wiener-SIM to reconstruct all the sub-blocks of Tiled-SIM, which is beneficial in removing artifacts and residual background fluorescence in Tiled-SIM reconstruction (**f**). Scale bar in (**a, b**) 8 μm; in (**c-f**), 1 μm.
